# Using mass spectrometry imaging to map fluxes quantitatively in the tumor ecosystem

**DOI:** 10.1038/s41467-023-38403-x

**Published:** 2023-05-19

**Authors:** Michaela Schwaiger-Haber, Ethan Stancliffe, Dhanalakshmi S. Anbukumar, Blake Sells, Jia Yi, Kevin Cho, Kayla Adkins-Travis, Milan G. Chheda, Leah P. Shriver, Gary J. Patti

**Affiliations:** 1 Department of Chemistry, Washington University in St. Louis, St. Louis, MO USA; 2 Center for Metabolomics and Isotope Tracing, Washington University in St. Louis, St. Louis, MO USA; 3 Department of Medicine, Washington University in St. Louis, St. Louis, MO USA; 4 Department of Neurology, Washington University in St. Louis, St. Louis, MO USA; 5 Siteman Cancer Center, Washington University in St. Louis, St. Louis, MO USA

**Keywords:** Metabolomics, Bioinformatics, Imaging, Mass spectrometry, Metabolism

## Abstract

Tumors are comprised of a multitude of cell types spanning different microenvironments. Mass spectrometry imaging (MSI) has the potential to identify metabolic patterns within the tumor ecosystem and surrounding tissues, but conventional workflows have not yet fully integrated the breadth of experimental techniques in metabolomics. Here, we combine MSI, stable isotope labeling, and a spatial variant of Isotopologue Spectral Analysis to map distributions of metabolite abundances, nutrient contributions, and metabolic turnover fluxes across the brains of mice harboring GL261 glioma, a widely used model for glioblastoma. When integrated with MSI, the combination of ion mobility, desorption electrospray ionization, and matrix assisted laser desorption ionization reveals alterations in multiple anabolic pathways. De novo fatty acid synthesis flux is increased by approximately 3-fold in glioma relative to surrounding healthy tissue. Fatty acid elongation flux is elevated even higher at 8-fold relative to surrounding healthy tissue and highlights the importance of elongase activity in glioma.

## Abstract (web summary)

Isotopologue spectral analysis was originally designed to assess metabolic fluxes from bulk samples. Here, the authors adapted this approach to infer fluxes from discrete regions in tissue by using mass spectrometry imaging, showing increased fatty acid synthesis flux in brain tumors of mice.

## Introduction

Recent technological and computational advances in spatial transcriptomics have increased interest in complementary techniques for characterizing the multi-dimensional architecture of tissues, such as using mass spectrometry imaging (MSI) to map the distribution of metabolites and lipids^1,2^. Unlike liquid chromatography/mass spectrometry (LC/MS), where metabolites and lipids are usually measured from whole samples in bulk, MSI uses a scanning probe to collect spatially resolved data across a tissue section. Although its chemical coverage is typically inferior to LC/MS, MSI still has the capability to measure metabolites and lipids with high sensitivity and molecular specificity^3^.

To perform MSI, molecules from tissue sections must be converted into gas-phase ions. Historically, this has been most commonly achieved by using Matrix Assisted Laser Desorption/Ionization (MALDI). More recently, however, instruments equipped with a Desorption Electrospray Ionization (DESI) source have also become commercially available and are being increasingly used. While it is possible to conduct MSI experiments with either MALDI or DESI, molecules can have different ionization efficiencies with each approach. Thus, depending on the analytes of interest, MALDI may prove to be more sensitive than DESI, or vice versa. MALDI and DESI also have unique strengths that might be better suited for specific applications or workflows. MALDI, for instance, requires that samples be co-crystallized with a chemical matrix. In addition to complicating sample preparation, matrix compounds produce peaks in the low-mass region of the data that can interfere with the detection of metabolites. DESI does not require a matrix but tends to have less spatial resolution than MALDI^4^.

Regardless of the ionization method used, to date, the majority of MSI studies interrogating spatial metabolism have focused on the relative levels of metabolites and lipids (also known as pool sizes). The impressive number of reports already published, highlights the important insights that such an approach can yield^3,4^. Notwithstanding, there are two notable barriers that limit interpretation of metabolite and lipid pool sizes as assessed by MSI. First, changes in the relative abundance of a compound are difficult to determine between different regions of a tissue section. Among the quantitative challenges is that, unlike LC/MS, MSI simultaneously subjects all of the molecules within a particular tissue location to the ionization process without any prefractionation^5^. Given that the abundance of other molecules in a sample can influence the ionization efficiency of the target analyte, changes in MSI signal intensity between tissue regions do not necessarily correspond to changes in the actual concentration of the target analyte^6^. This is particularly a concern when comparing sample regions of diverse composition, such as tumor and healthy tissue, where a large number of molecular differences may contribute to variable matrix effects. The second limitation of pool size-based MSI studies is that metabolite abundances do not fully capture the dynamics of metabolism^7^. Metabolic pathway fluxes cannot be reliably inferred from metabolite concentrations alone^8^. Similarly, even when the concentration of a metabolite remains constant, the precursor from which it is derived might change^9^.

Combining stable isotope tracers with MSI can help address both of the aforementioned challenges. In a typical stable isotope labeling experiment, a biological system is administered a nutrient that contains a rare isotope such as ^13^C. Mass spectrometry can then be used to measure isotopologues, which are molecules that only differ by their isotopic composition. Heavy isotopologues appear in metabolites downstream of the labeled nutrient as they incorporate rare isotopes from the tracer^10^. Biochemical pathway activities can be inferred from a combination of the pattern of isotopologue species present and the rate at which they appear after administration of the tracer^11^. Notably, because isotopologues of a metabolite have the same ionization efficiency, differences in the physicochemical properties across a tissue will not confound quantitative assessment of metabolite labeling, unlike metabolite pool sizes^12,13^. Despite the appeal, only a limited number of studies have directly administered labeled compounds to animals in vivo before collecting tissue for MSI of intact molecules. The majority of these studies have restricted analysis to the localization of labeled metabolites^14,15^. Only a few have started to extract the metabolic information encoded by per-pixel isotopologue calculations^16–25^, and workflows for flux analysis have not yet been established.

Here, we sought to expand upon MSI approaches using stable isotope tracers by employing a combination of MALDI, DESI, and ion mobility spectrometry (IMS) to study glioblastoma, which is the most common type of malignant brain tumor among adults^26^. We applied a widely used syngeneic C57BL/6 model of glioblastoma generated by intracerebral injection of GL261 cells harboring a R132H mutation in isocitrate dehydrogenase 1 (IDH1). Prior studies have demonstrated that GL261 tumors exhibit genetic, histologic, and vascular heterogeneity^27–29^, but potential changes in metabolic activity throughout the tumor ecosystem have not been well characterized. Moreover, the impact that tumors have on the metabolism of surrounding brain regions comprised of healthy tissue remains poorly understood. In this work, we aimed to investigate the metabolic architecture of brains harboring glioma by combining stable isotope labeling and MSI. We developed an approach that we refer to as Spatial Isotopologue Spectral Analysis (SISA) to assess fractional fluxes of fatty acid synthesis and elongation on a per-pixel basis. While absolute fluxes are presented in units of moles per time^30^, fractional fluxes are unitless and describe the fraction of a metabolite pool that has been newly synthesized during the labeling period^31^. Fractional fluxes determined by SISA enable metabolic pathway activities to be quantitatively compared between different conditions^32^. In the current study, mapping fractional fluxes throughout the brain revealed biochemical pathways that are uniquely activated in tumor tissue and provided an assessment of the  degree of metabolic homogeneity in our model of glioblastoma.

## Results

### Approach for imaging with stable isotope tracers

**Fig. 1:**
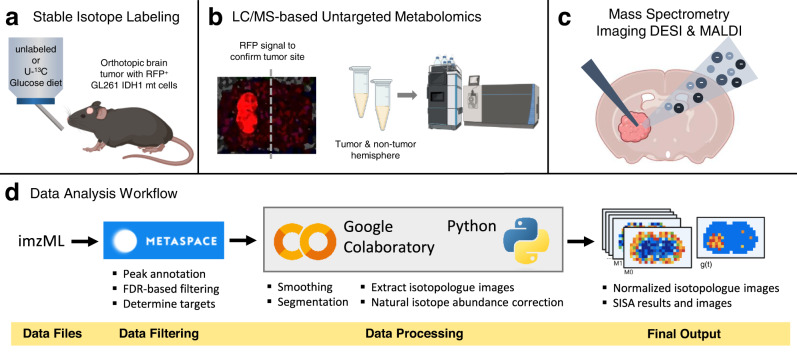
Mass spectrometry imaging with stable isotope labeling workflow. **a** Mice with orthotopic brain tumors were fed an unlabeled or U−^13^C glucose-based liquid diet for 48 h before being imaged. **b** Collected brains were cryo-sectioned and the tumor site was confirmed with fluorescence microscopy by using the red fluorescent protein (RFP) expressed by the implanted GL261 IHD1 mutant cells. Tumor and non-tumor hemisphere were analyzed by LC/MS, which revealed differences in metabolite levels. **c** Mass spectrometry imaging analysis of unlabeled and labeled tumor brain sections by DESI and MALDI. **d** Data analysis workflow. Vendor-specific data files are first converted into the open-source imzML format. METASPACE^34^ (https://metaspace2020.eu) is then used to annotate peaks on the basis of accurate mass and natural abundance isotope patterns. FDR-based filtering enables determination of reliable target metabolites. The data are then processed in a targeted way through the use of a Python package that we developed to process MSI data. The software can be run online within Google Colaboratory, which does not require any local installation of Python or related packages. The output of the analysis software is normalized isotopologue images (corrected for natural isotope abundance). Additionally, Spatial Isotopologue Spectral Analysis (SISA) can be performed on each pixel to yield the fractional biosynthetic flux term, *g(t)*.

We applied an integrated methodology for this study that leverages MSI, IMS, and LC/MS to analyze brain tissue. To generate samples, mice with intracerebral implantation of murine glioma GL261 IDH1 mutant cells were fed a liquid diet containing unlabeled or U-^13^C glucose for 48 h before harvesting tissue as described previously (Fig. 1a)^33^. Central carbon metabolites reach isotopic steady state within this time range^33^. GL261 mutant cells were engineered to express red fluorescent protein (RFP), which allowed us to confirm the tumor location in sections adjacent to those imaged by DESI and MALDI or analyzed by LC/MS (Fig. 1b, c). Beyond FDR-controlled metabolite annotation with METASPACE^34^, compound identification is supported by both collisional cross section (CCS) values from IMS in imaging mode and retention times from LC/MS. In this way, we benchmarked MSI results against those from established LC/MS methods, with the limitation that the LC/MS data collapsed spatial information into an average across bulk tissue.

Accurate quantitation of isotope enrichment is essential to interpret data from labeled samples. This requires that individual isotopologues be resolved from other signals. In tracer studies performed with LC/MS, chromatographic separation helps to remove compounds that might overlap with isotopologues. In MSI, however, all of the compounds that are ionized from a given pixel appear in the same mass spectrum. To ensure that isotopologue peaks were not contaminated with interferences in our analysis, we used IMS and unlabeled control samples. IMS facilitates detection of interferences that have different CCS values. Unlabeled control samples enable identification of signals that have overlapping *m/z* values with isotopologues of interest. When a signal that had an *m/z* value overlapping with an isotopologue peak appeared in the unlabeled control sample, we assumed that particular isotopologue could not be accurately measured from labeled samples and excluded it from our analysis. As expected due to the intrinsic differences in ionization techniques and matrix peaks, some signals from brain tissue were only detected by our DESI method and others were only detected by our MALDI method (Supplementary Fig. 1). These differences resulted in a unique set of interferences with each platform, which influenced our ability to resolve isotope labeling patterns. To validate that our conclusions were not affected by such interferences, we performed all experiments by using both DESI and MALDI MSI.

To analyze our results, we developed an open-source, Python-based platform for analysis of MSI data from isotopically labeled tissues that includes methods for image preprocessing (filtering, segmentation, normalization, etc.), isotopologue image extraction, ^13^C natural isotope abundance correction, and flux analysis. As shown by both DESI and MALDI results, our platform is compatible with MSI data generated with different modalities and accepts the open MSI data format imzML^35^. To create isotopologue images, we first extracted the signal for each detected metabolite isotopologue. Summing the signals of all isotopologues for each metabolite provides an estimate of relative pool size distributions, which is the output of conventional MSI experiments that do not involve stable isotope labeling. Next, we created fractional isotopologue images by dividing each isotopologue image by the corresponding summed isotopologue image for a particular metabolite (Fig. 1d). Fractional isotopologue images are a per-pixel plot of fractional labeling,$\,{f}_{x}$, defined as the abundance of an individual isotopologue, ${a}_{x}$, divided by the sum of all isotopologues for the metabolite that contains $n$ carbon atoms as shown in Eq. 1^36^.

1 $${f}_{x}=\frac{{a}_{x}}{{\sum }_{j=0}^{n}{a}_{j}}$$

A complete description of the data processing procedures developed and applied in this study is included in the “Methods” section.

In addition to the intrinsic advantage of canceling out pixel-specific ion suppression, fractional isotopologue images provide spatially resolved insights into metabolic dynamics. Interpretation of isotopologue fractions allow for spatial indexing of: (i) nutrient utilization, (ii) pathway activities that produce specific isotopologue patterns, and (iii) flux as assessed by performing SISA to measure fatty acid turnover rates. We present these data after natural isotope abundance correction together with the summed isotopologue images to highlight the different information content available from the same sample for the conventional and isotope labeling imaging domains.

### Untargeted metabolomics identifies differences between tumor and non-tumor brain hemispheres

**Fig. 2:**
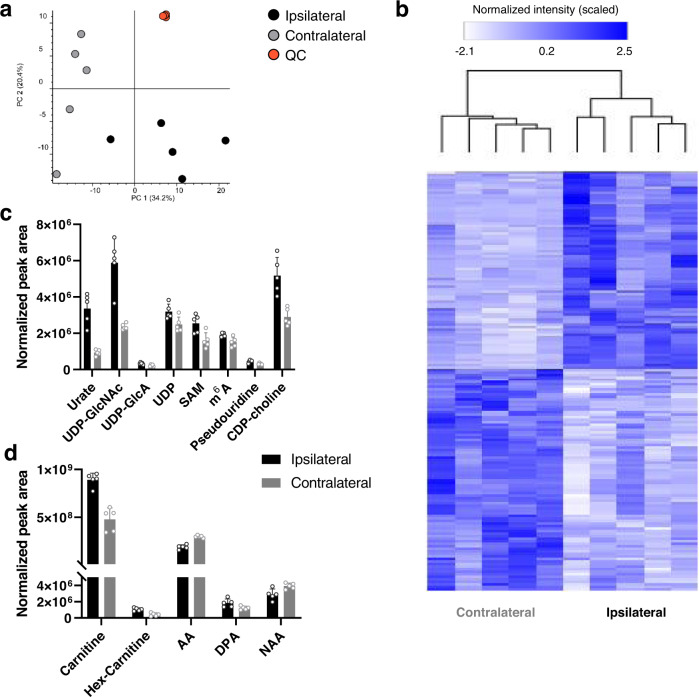
Untargeted LC/MS-based metabolomics of unlabeled brains. Ipsilateral (tumor) and contralateral (non-tumor) hemispheres were extracted and analyzed by using untargeted metabolomics. **a** Principal component analysis of all detected compounds with a CV of less than or equal to 20% in at least one of the two sample groups show separation of the ipsilateral (black) and contralateral (gray) hemispheres, with tight clustering of the quality-control samples (orange). **b** Heatmap of metabolites after filtering based on a CV of ≤20% in at least one of the two sample groups, and a *p* value ≤ 0.05 (not corrected for multiple hypothesis testing). **c** Nucleotide-related metabolites, **d** fatty acid-related metabolites and *N*-acetylaspartate (NAA) show alterations between the two brain hemispheres. Data are from five unlabeled mouse brains, **c** + **d** show means ± SDs of five unlabeled mice. UDP-GlcNAc uridine diphosphate-*N*-acetylglucosamine, UDP-GlcA uridine diphosphoglucuronate, UDP uridine diphosphate, SAM S-Adenosylmethionine, m6A *N*^6^-Methyladenosine, CDP-choline cytidine 5′-diphosphocholine, AA arachidonate, DPA docosapentaenate. Source data are provided as a Source Data file.

For LC/MS-based untargeted metabolomics, 50 µm coronal slices of brain tissue from mice administered an unlabeled glucose-based liquid diet were sectioned into the ipsilateral (tumor-containing) and the contralateral (tumor-free) hemispheres. Metabolites from each hemisphere were extracted separately. After grouping of redundant features and removal of background, our analysis resulted in 459 compounds with a coefficient of variation (CV) of ≤20% in at least one of the two sample groups (see “Methods” section for details). Performing principal component analysis on these compounds showed clear clustering of the contralateral and ipsilateral tissues (Fig. 2a). Relative to the non-tumor hemisphere, we found 78 metabolites with elevated pool sizes and 88 metabolites with decreased pool sizes (*p*-value < 0.05) in the ipsilateral tissue (Fig. 2b). Among these altered compounds were nucleotide-related metabolites such as urate and UDP-*N*-acetylglucosamine, fatty acids, and *N*-acetylaspartate (NAA, Fig. 2c, d). The major limitation of LC/MS-based approaches such as the one applied here is that, because tissues are analyzed in bulk, these methods cannot determine whether metabolites are altered in the tumor region itself or in surrounding tissue. Furthermore, by only evaluating metabolite pool sizes, an incomplete picture of metabolism is obtained. To better understand metabolic dynamics, such as whether the level of an intermediate is elevated due to increased production or decreased consumption of a metabolite, stable isotopes are needed. Accordingly, we assessed tissues from animals fed ^13^C-enriched diets by MSI and LC/MS to obtain more insight into metabolic changes associated with glioma.

### NAA imaging reveals increased TCA cycle cataplerosis in the tumor region

**Fig. 3:**
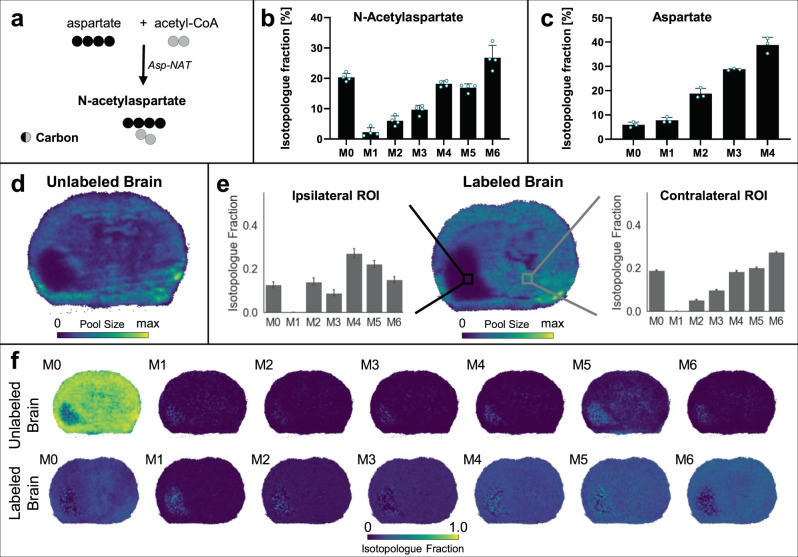
N-Acetylaspartate (NAA) isotopologue imaging with DESI. **a** Aspartate *N*-acetyltransferase (Asp-NAT) synthesizes NAA from aspartate and acetyl-coenzymeA (acetyl-CoA). Black and gray circles denote carbons derived from different molecules. **b** + **c** LC/MS isotopologue fractions from labeled tumor brains show enrichment of high-mass isotopologues for **b** NAA and **c** aspartate. **d** DESI imaging shows total NAA pool size in an unlabeled tumor brain. **e** DESI imaging of NAA pool size (from summing the intensity of all isotopologues except M1) in the labeled tumor brain, with two regions of interest (ROI) selected for comparison of NAA labeling patterns. The ROI contralateral to the tumor shows a higher M6 compared to the ROI in the tumor region (ipsilateral). The same pool size trends can be seen in the unlabeled and labeled brain. **f** Isotopologue fractional images of NAA for both an unlabeled (top) and a labeled (bottom) tumor brain. Labeling patterns have been natural isotope abundance corrected. Due to the low intensity of NAA in the tumor region, small fluctuations in the baseline measured from unlabeled brain tissue lead to apparent M5 and M6 signal. To validate that these signals are noise and do not affect our biological conclusions, we repeated the analysis with MALDI where NAA signal intensity is higher and no background in unlabeled brain is detected. The MALDI counterpart from two additional brains is shown in Fig. 4. Data in **b** + **c** are shown as means ± SDs for *n* = 4 (NAA) and *n* = 3 (aspartate) biological replicates. The isotopologue plots in **e** show data from all pixels within the ROI (>100) as mean ± SD. Source data are provided as a Source Data file.

First, we wanted to demonstrate that our approach provides unique information compared to conventional imaging of metabolite pool sizes. NAA is one of the most abundant small molecules in the brain, where it is synthesized from aspartate and acetyl coenzyme A (acetyl-CoA, Fig. 3a)^37^. Among other metabolic fates, NAA can be used as a precursor to synthesize *N*-acetylaspartylglutamate or it can be degraded to provide a source of acetyl-CoA^38,39^. NAA synthesis has been best studied in neurons and is a known marker of neuronal health^38^. Of note, NAA has also been shown to be produced by GL261 cells in vitro^40^. Indeed, when the GL261 cells used in this study were cultured in labeled glucose, we observed production of labeled NAA (Supplementary Fig. 2). Evaluation of labeled brain tissue by LC/MS showed that the most abundant isotopologue of NAA is M6, indicating that much of the NAA pool is derived from fully labeled aspartate and acetyl-CoA (Fig. 3b). Consistent with this result, we found that nearly 40% of aspartate incorporated four ^13^C-labels from glucose (Fig. 3c). When brain tissue was analyzed with DESI-IMS mass spectrometry, putative NAA isotopologues co-migrated (except for M1), and CCS data closely matched reference values (Supplementary Fig. 3). Assessment of migration times increased our confidence in metabolite identification and signified that the M1 of NAA is contaminated with an interfering ion.

DESI MSI images of total NAA intensities were generated from unlabeled (Fig. 3d) and labeled (Fig. 3e) brains, both harboring gliomas. For animals fed U-^13^C glucose, images were obtained by summing all isotopologues of NAA, except for M1. Summing the isotopologues collapses the data into the same information domain yielded by experiments without stable isotope labeling. Fluorescence microscopy was used to confirm the location of the tumor (Supplementary Fig. 4). The results show lower NAA signal in the tumor relative to other tissue regions, which is consistent with a decreased local concentration of NAA and reduced neuronal health/populations in the tumor region.

From the brains of mice administered isotope tracers, isotopologue plots can be extracted from regions of interest (ROIs), as shown in Fig. 3e. In addition to comparing fractional labeling from ROIs, we found it useful to consider fractional carbon-atom labeling. Fractional labeling and fractional carbon-atom labeling are calculated differently and provide unique information. Fractional labeling is determined by Eq. 1 and describes the amount of a metabolite that has a given number of ^13^C-labels (e.g., the fraction of a metabolite with M2 labeling). Fractional labeling is useful to compare the relative activities of biochemical pathways that produce different isotopologues^41^. Fractional carbon-atom labeling ($L$), on the other hand, describes the fraction of carbon in a metabolite that is ^13^C-labeled according to Eq. 2.

2 $$L=\,\frac{{\sum }_{j=0}^{n}j\cdot {a}_{j}}{n{\sum }_{j=0}^{n}{a}_{j}}$$

Fractional carbon-atom labeling has been used to assess the total carbon contribution of a uniformly ^13^C-labeled tracer to a given metabolite^42^. When comparing two ROIs, indicated by a black square for the tumor region and a gray square for the contralateral non-tumor region in Fig. 3e, we found differences in the fractional carbon-atom labeling from glucose carbon to NAA synthesis. We determined that the fractional carbon-atom labeling was lower in the ipsilateral ROI (41.8%) compared to the contralateral ROI (51.8%). As the M1 isotopologue of NAA was contaminated, this isotopologue was excluded from the fractional carbon-atom labeling calculation. A per-pixel analysis of isotopologue fractional abundances also showed more M6 labeling in the non-tumor region compared to the tumor region (Fig. 3f). These data are consistent with increased cataplerosis in the tumor region, with TCA cycle intermediates being used to synthesize macromolecules required for proliferation before they can undergo multiple turns of ^13^C-label incorporation.

**Fig. 4:**
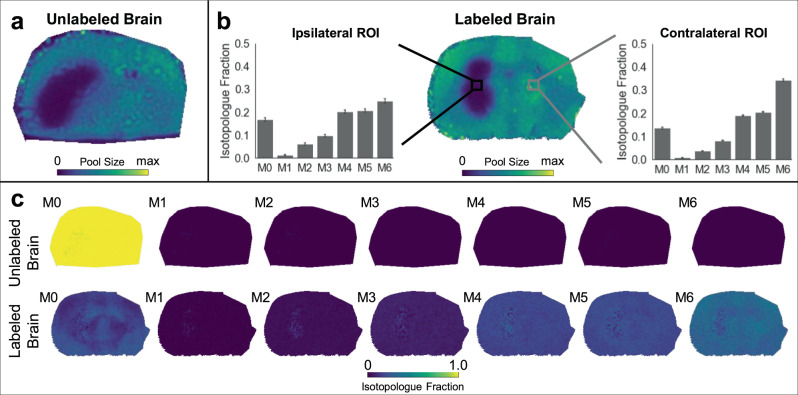
*N*-Acetylaspartate (NAA) isotopologue imaging with MALDI. **a** MALDI imaging shows total NAA pool size in an unlabeled tumor brain. **b** NAA pool size in the labeled tumor brain, with two regions of interest (ROI) selected for comparison of NAA labeling patterns. The ROI contralateral to the tumor shows a higher M6 compared to the ROI in the tumor region (ipsilateral). The same pool size trends can be seen in the unlabeled and labeled brain. **c** Isotopologue fractional images of NAA for both an unlabeled (top) and a labeled (bottom) tumor brain. Labeling patterns have been natural isotope abundance corrected. The unlabeled brain shows no signal for labeled isotopologues. The M6 in the labeled brain shows a decrease in the tumor region, as expected from the extracted ROIs. The DESI counterpart from two additional brains is shown in Fig. 3. The isotopologue plots in **b** show data from all pixels within the ROI (>100) as mean ± SD.

A challenge of imaging NAA by DESI is that its low level in the tumor region is near the limit of detection. Although the average isotopologue fractions for NAA were similar when assessed by DESI and LC/MS (Supplementary Fig. 5), we are unable to validate the spatial labeling patterns obtained from DESI with LC/MS alone. Hence, to provide additional confidence in our images of isotopologues fractional abundances, we analyzed the same brain tissues by MALDI (Fig. 4). Note that the brain samples shown in the main figures are from separate mice. We point out that, when comparing DESI to MALDI, different metabolites can ionize with different efficiencies^4^. For NAA specifically, MALDI analysis with *N*-(1-Naphthyl) ethylenediamine dihydrochloride (NEDC) matrix proved to be superior because it had better sensitivity and no interfering ions were measured from unlabeled brains. Our MALDI results confirmed depleted levels of NAA in the tumor (Fig. 4a) and increased M6 NAA labeling in non-tumor regions (Fig. 4b, c) that indicate impaired neuronal health and increased TCA cycle cataplerosis.

### Adenosine monophosphate imaging highlights changes in purine biosynthesis

**Fig. 5:**
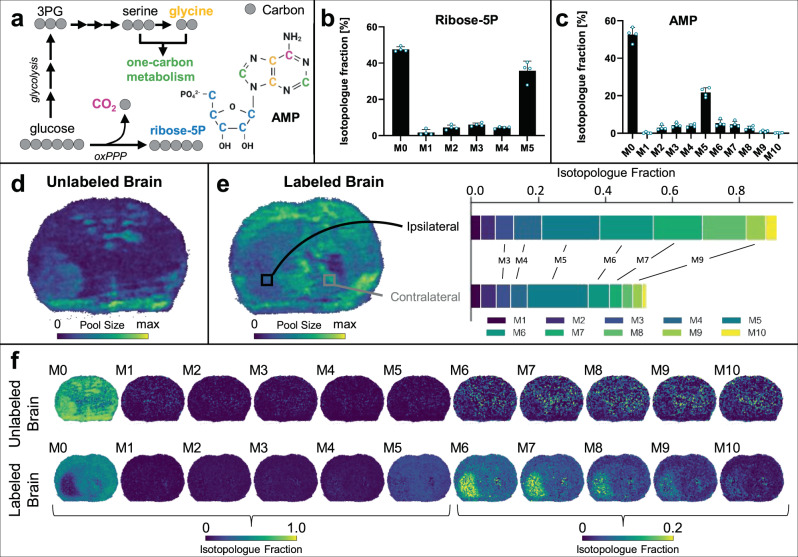
Adenosine monophosphate (AMP) isotopologue imaging with DESI. **a** Glucose is converted to ribose 5-phosphate (R5P) via the oxidative pentose phosphate pathway (oxPPP). Glucose also provides a source of serine, glycine, and CO_2_ (one of which is shown). R5P, serine, glycine, and CO_2_ can be used for purine synthesis to make inosine- monophosphate, which is then converted to AMP. **b** + **c** LC/MS isotopologue fractions from labeled tumor brains show high M5 enrichment of **b** R5P and **c** AMP isotopologues. **d** DESI imaging shows the total AMP pool size in an unlabeled tumor brain. **e** DESI imaging of AMP pool size in the labeled tumor brain. Two regions of interest (ROI) selected for comparison of AMP labeling patterns show higher enrichment in heavier isotopologues from the tumor region. **f** Isotopologue fractional images of AMP for both the unlabeled (top) and labeled (bottom) brains. Labeling patterns have been natural isotope abundance corrected. M5 R5P shows a consistent distribution across the whole brain but ^13^C labeling of higher isotopologues in the tumor region point to increased usage of glucose for purine synthesis. Note the different intensity scale for M6 to M10 compared to the other isotopologues. Given the low level of signal intensity being plotted for M6 to M10, small fluctuations in the baseline lead to some noise in unlabeled brain. We note that the patterns we observe in the labeled brain are specific to the tumor region, indicating that they are not a result of noise. Nonetheless, to validate our biological conclusions, we repeated the experiment with MALDI where no background in unlabeled brain is detected. The MALDI counterpart from two additional brains is shown in Fig. 6. Data in **b** + **c** are shown as means ± SDs for *n* = 4 biological replicates. Source data are provided as a Source Data file.

Next, we wanted to extend our analysis to probe other aspects of tumor metabolism on a per-pixel basis. Adenosine monophosphate (AMP) was an attractive target analyte because its labeling pattern simultaneously encodes information related to the pentose phosphate pathway (PPP) and one-carbon metabolism (Fig. 5a). AMP contains ten carbons, five of which come from ribose and five of which come from a purine base. As shown in Fig. 5b, the ribose can be derived from glucose via the oxidative PPP. The purine carbons can also be derived from glucose (Fig. 5c). One of the purine carbons comes from bicarbonate, which can originate from the decarboxylation of glucose. The remaining carbons can come from serine and glycine through a series of reactions starting with the glycolytic intermediate 3-phosphoglycerate and involving one-carbon metabolism. These pathways provide biochemical routes for producing each isotopologue of AMP (M1-M10) from the tissues of animals fed U-^13^C glucose diets.

**Fig. 6:**
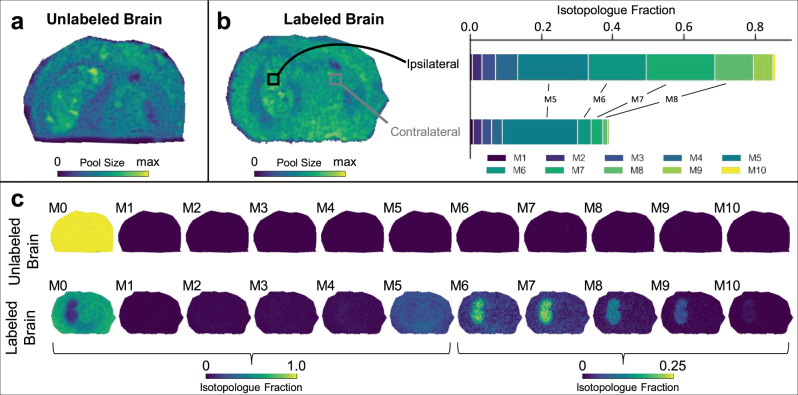
Adenosine monophosphate (AMP) isotopologue imaging with MALDI. **a** MALDI imaging shows the total AMP pool size in an unlabeled tumor brain. **b** AMP pool size in the labeled tumor brain. Two regions of interest (ROI) selected for comparison of AMP labeling patterns show higher enrichment in heavier isotopologues from the tumor region. **c** Isotopologue fractional images of AMP for both the unlabeled (top) and labeled (bottom) brains. Labeling patterns have been natural isotope abundance corrected. Note the different intensity scale for M6 to M10 compared to the other isotopologues. The DESI counterpart from two additional brains is shown in Fig. 5.

We first evaluated AMP in unlabeled (Fig. 5d) and labeled (Fig. 5e) glioma brains by using DESI MSI. Notably, unlabeled samples did not produce any major interferences with AMP isotopologues (Fig. 5f), and IMS analysis indicated co-migration of AMP isotopologues in labeled specimens. Moreover, the CCS data of AMP closely matched reference values (Supplementary Fig. 3b). Examination of the isotopologue patterns from AMP revealed diffuse M5 labeling across the tissue and indicated oxidative PPP activity throughout the brain, which is consistent with prior work suggesting that the oxidative PPP is essential for healthy brain function^43^. In contrast to the pattern of M5, we found that isotopologues M6-M9 were uniquely elevated in glioma compared to healthy tissue (Fig. 5f). When we repeated the analysis by using MALDI, which had better signal to noise for AMP than DESI, we observed comparable patterns in the fractional isotopologue images (Fig. 6). MSI labeling data averaged from the whole brain were also in agreement with LC/MS data from bulk tissue (Supplementary Fig. 5). The results demonstrate that more AMP is synthesized from glucose in glioma compared to healthy tissue. Higher M6-M9 labeling of AMP in the tumor indicates that more glucose is used for de novo purine biosynthesis in that region, with glucose providing an increased contribution to glycine and carbon units for one-carbon metabolism. These findings are consistent with cancer cells having an increased demand for one-carbon units to synthesize nucleotides that are needed for proliferation, as has been suggested previously^44^. It is interesting to point out that de novo pyrimidine synthesis has recently been described as a potential target for IDH mutant gliomas^45^ and IMP dehydrogenase-2 has been found to promote tumorigenesis in glioblastoma^46^.

### Mapping de novo lipogenesis flux

**Fig. 7:**
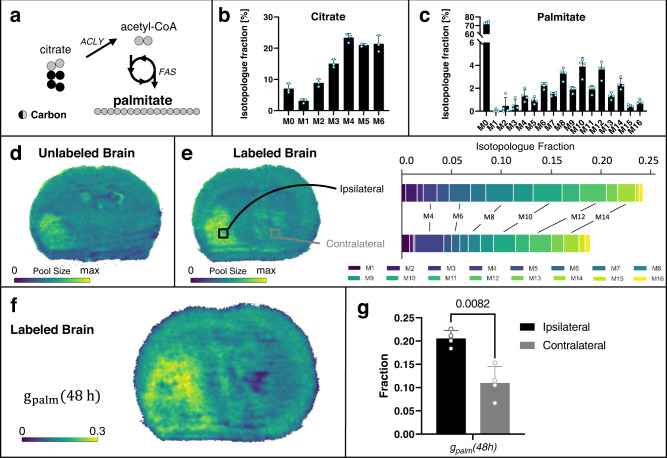
Palmitate (palm) isotopologue imaging with DESI. **a** Acetyl-CoA generated from citrate by ATP-citrate lyase (ACLY) is utilized by fatty acid synthase (FAS) for de novo palmitate synthesis. Black and gray circles denote carbons derived from different molecules. **b** + **c** LC/MS isotopologue fractions from labeled tumor brains show enrichment of high mass isotopologues of **b** citrate and **c** palmitate in the bulk tissue, suggesting acetyl-CoA derived from citrate for fatty acid synthesis will be enriched in ^13^C. **d** DESI imaging shows palmitate pool size in unlabeled tumor brain. **e** DESI imaging of labeled tumor brain indicates palmitate pool size, with two regions of interest (ROI) selected for comparison of palmitate labeling patterns. The tumor (ipsilateral) region shows higher labeling than the non-tumor (contralateral) region, with both ROIs having higher enrichment in even isotopologues. **f** Flux image after applying SISA to the labeled tumor brain shows the palmitate turnover, *g*_*palm*_*(48* *h)*, is higher in the tumor region. **g** Comparison of *g*_*palm*_*(48* *h)* in the tumor site versus non-tumor tissue in four biological replicates. Data shown as mean ± SD. *p* value from paired *t* test (two-tailed). Lipogenic flux images for all four biological replicates with corresponding microscopy images are shown in Supplementary Fig. 10. The MALDI counterpart from two additional brains is shown in Fig. 8. Data in **b** + **c** are shown as means ± SDs for *n* = 3 (citrate) and *n* = 4 (palmitate) biological replicates. Source data are provided as a Source Data file.

Palmitate, and other lipids derived from it, are essential for proliferating cells to form new membranes. Here, we wished to evaluate whether glioma fulfill their lipid needs by de novo lipogenesis or by consuming exogenous lipids from the environment. Given that palmitate is the major end product of fatty acid synthase, it is well suited to assess the rate of de novo lipogenesis in cells and tissues^47^. Palmitate is synthesized in the cytosol from eight acetyl-CoA molecules that are primarily derived from citrate via ATP-citrate lyase (Fig. 7a). As expected, LC/MS analysis shows that citrate and palmitate incorporate glucose carbon (Fig. 7b, c).

**Fig. 8:**
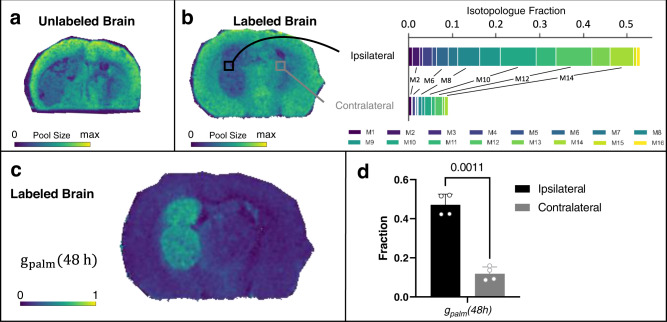
Palmitate (palm) isotopologue imaging with MALDI. **a** MALDI imaging shows palmitate pool size in unlabeled tumor brain. **b** Palmitate pool size of labeled tumor brain, with two regions of interest (ROI) selected for comparison of palmitate labeling patterns. The tumor (ipsilateral) region shows higher labeling than the non-tumor (contralateral) region, with both ROIs having higher enrichment in even isotopologues. **c** Flux image after applying SISA to the labeled tumor brain shows that palmitate turnover, *g*_*palm*_*(48* *h)*, is higher in the tumor region. **d** Comparison of *g*_*palm*_*(48* *h)* in the tumor site versus non-tumor tissue in four biological replicates. Data shown as mean ± SD. *p* value from paired t test (two-tailed). Lipogenic flux images for all four biological replicates with corresponding microscopy images are shown in Supplementary Fig. 11. The DESI counterpart from two additional brains is shown in Fig. 7. Source data are provided as a Source Data file.

We first used DESI to image palmitate from the brains of unlabeled and labeled mice harboring glioma (Fig. 7). Data from IMS and unlabeled samples demonstrated that the M1, M4, and M5 of palmitate had interferences, requiring that these isotopologues be excluded from our DESI analysis (Supplementary Figs. 3c and 6). To ensure that the exclusion of these isotopologues did not affect the results, we repeated the analysis by using MALDI to image palmitate from the brains of glioma mice (Fig. 8). Unlike DESI, no major interferences were observed for palmitate isotopologues in our MALDI data (Supplementary Fig. 7). Interestingly, DESI and MALDI provided inconsistent pool-size results for palmitate. This included pool sizes assessed from unlabeled tissues and those obtained by summing palmitate isotopologues (ignoring M1, M4, and M5 in DESI data). With DESI, palmitate signal was elevated in the tumor relative to healthy tissue regions (Fig. 7d, e and Supplementary Fig. 6). In MALDI experiments, on the other hand, palmitate signal was depleted in the tumor relative to other brain regions (Fig. 8a, b and Supplementary Fig. 7). Meanwhile, the pool size of palmitate between the tumor and non-tumor hemispheres was the same when measured by LC/MS (see Supplementary Fig. 5). These data underscore that variable ionization effects between tissue regions of different composition can make pool-size data unreliable. We wish to emphasize that relative pool-size distributions have no effect on our ability to interpret labeling data. Analysis of labeling data only requires quantitation of isotope enrichment, which are not influenced by matrix effects. Indeed, when data from whole-brain tissue were averaged together, the same trend in palmitate isotopologue abundances was observed from DESI, MALDI, and LC/MS (Supplementary Fig. 5). The only notable difference was that isotopologues from DESI were lower due to background palmitate from DESI slides (Supplementary Fig. 8). When considering endogenous labeling, background palmitate signal merely creates a dilution factor and does not impede comparisons of relative fluxes between tumor and healthy tissue. In both DESI and MALDI images, palmitate had higher labeling in the tumor compared to other regions.

Although isotopic steady state was achieved in central carbon metabolites within the 48 hours that mice were fed U-^13^C glucose, isotopic steady state was not reached for longer-lived metabolites such as palmitate and other fatty acids. Flux analysis for isotopically nonstationary pathways typically uses multiple time points to characterize the dynamics of label incorporation, which enables flux to be inferred through the construction of ordinary differential equations^48^. For some isotopically nonstationary metabolites such as those synthesized as homopolymers, however, fractional label incorporation encodes flux information that is recoverable by using specialized models even from a single time point^31,49^. Given that all sixteen carbons in palmitate are derived from the acetyl group of eight acetyl-CoA precursors, we sought to apply a spatial variant of such a specialized model known as isotopologue spectral analysis (ISA, also known as isotopomer spectral analysis) to map de novo lipogenesis fractional turnover flux on a per-pixel basis across a single MSI dataset.

The standard ISA approach models the isotopologue distribution of a biopolymer as a function of: (i) the fractional contribution from labeled compound to the biosynthetic monomer pool, *D*, and (ii) polymer flux, represented as fractional turnover within the labeling time, *g(t)*^31,49^. This enables the biosynthesis rate to be separated from nutrient utilization. Derived from eight acetyl-CoA monomers, the palmitate isotopologue fractional distribution represents an eighth-order polynomial in the ISA model. Each possible isotopologue fractional distribution for palmitate can be evaluated to determine a unique set of *D* and *g(t)* values. Conventionally, applying the ISA model requires known isotopic labeling in the tracer monomer pool, which is acetyl-CoA derived from U-^13^C glucose here^31^. The total biosynthetic pool utilized to synthesize palmitate is then represented as the fraction of the acetyl-CoA pool that originates from the tracer (*D*) and the isotopic labeling of acetyl-CoA that is derived from glucose. None of the mass spectrometry methods that we applied in this study enabled direct measurement of acetyl carbon labeling in acetyl-CoA. While cells labeled in culture with U-^13^C glucose mostly produce acetyl-CoA in which the acetyl group is M0 or M2, the M1 species tends to be more prevalent in vivo, possibly due to increased malic enzyme activity or recycling of endogenous CO_2_^33,50–52^. Indirect evidence of M1 and M2 acetyl-CoA in our animals is provided by citrate and palmitate labeling as measured by LC/MS from bulk tissue (Fig. 7b, c). Moreover, in our experiments, the different anatomical regions of the tissue will have altered glucose metabolism. As the labeling of acetyl-CoA is dependent on the activity of multiple pathways upstream of acetyl-CoA, we therefore cannot know or assume a single acetyl-CoA labeling pattern for use with the standard ISA approach. As an alternative, we developed a modified version of ISA that we refer to as SISA. Instead of inferring *D* from fixed tracer labeling, we infer the isotopic labeling of the entire monomer pool directly along with *g(t)* at each pixel. Thus, calculated *g(t)* rates are independent of each animal’s tracer uptake. This modification to ISA, while introducing an additional unknown to infer, does not reduce the accuracy or increase the sensitivity of the flux analysis to noise when compared to conventional ISA (Supplementary Fig. 9). Further details on SISA can be found in the Methods.

After applying SISA to the labeled palmitate MSI data, the fractional turnover flux at 48 h, *g*_*palm*_*(48* *h)*, was calculated at each pixel (Figs. 7f and 8c). Data from both DESI and MALDI show that palmitate turnover is significantly higher in tumor compared to other tissue. When using data from MALDI, which is less diluted by unlabeled palmitate background signal, we observe that ~55% of the observed palmitate pool for the tumor was synthesized within the 48 h timescale of tracer administration, while only ~20% was newly synthesized in non-tumor tissue. Replicate data for three additional labeled mice are shown in Supplementary Figs. 10 and 11. Statistical results for the four labeled mice are shown in Figs. 7g and 8d. To ensure these differences were not due to propagated measurement errors, we performed an error analysis and found that the expected absolute *g*_*palm*_*(48 h)* error is 0.008 ± 0.011 while our measured *g*_*palm*_*(48 h)* values range from 0.1 to 0.3 (see Supplementary Fig. 9). This analysis demonstrates that technical error is not likely to be a large contributor to the observed spatial variation. As validation, we also compared *g*_*palm*_*(48* *h)* and the calculated distribution of acetyl-CoA isotopologues from MSI data with the ISA results from the LC/MS analysis of corresponding extracted tissue and found the same trends (Supplementary Fig. 12).

From MSI data alone, we cannot rule out the possibility that palmitate is synthesized in another tissue (e.g., the liver) and subsequently transported to the brain where it is preferentially consumed by the tumor. Disruption of the blood-brain barrier within the tumor, for example, could make circulating lipids more accessible to glioma and potentially contribute to differential labeling patterns at the tumor site^53^. We expected that, if increased labeling of palmitate in glioma was mainly a result of tumor consuming labeled lipid from the circulation, the labeling of lipid in plasma would match the labeling of lipid in the tumor. By using palmitoylcarnitine as a surrogate for palmitate labeling to avoid potential palmitate background^47^, we compared the isotopologue patterns in serum to those in brain and found notable differences (Supplementary Fig. 13). We conclude that increased de novo lipogenesis in glioma is due to increased local fatty acid synthesis flux rather than increased uptake.

### Mapping fatty acid elongation flux

**Fig. 9:**
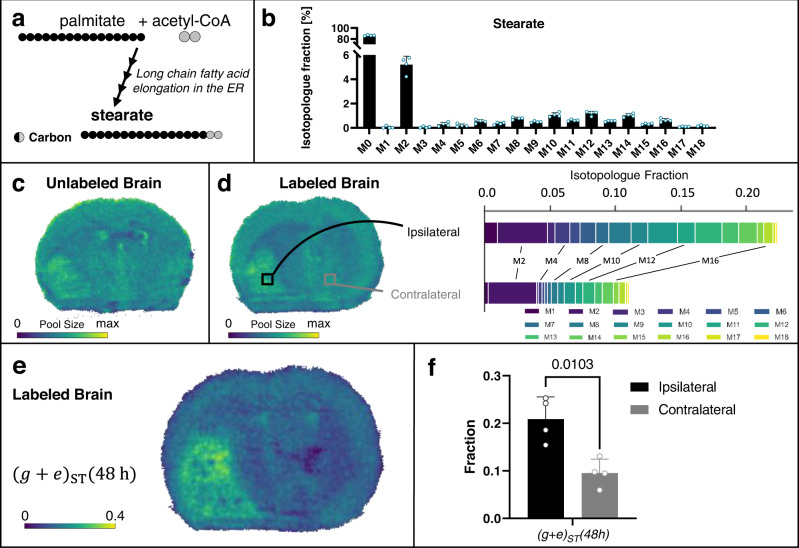
Stearate isotopologue imaging with DESI. **a** Acetyl-CoA is utilized by enzymes in the membrane of the endoplasmic reticulum (ER) for elongation of palmitate. **b** LC/MS isotopologue fractions from labeled tumor brains show enrichment of high mass isotopologues of stearate in the bulk tissue. **c** DESI imaging shows stearate pool size in the unlabeled tumor brain. **d** DESI imaging of the labeled brain indicates stearate pool size, with two regions of interest (ROI) selected for comparison of stearate labeling patterns. The tumor (ipsilateral) region shows higher labeling than the non-tumor (contralateral) region. **e** Flux image after applying SISA to a labeled tumor brain shows that the distribution of stearate turnover, *(g* + *e)*_*ST*_*(48* *h)*, is higher in the tumor region. **f** Comparison of *(g* + *e)*_*ST*_*(48* *h)* in the tumor site versus non-tumor tissue in four biological replicates. Data shown as mean ± SD. *p* value from paired t test (two-tailed). Elongation flux images for all four biological replicates with corresponding microscopy images are shown in Supplementary Fig. 10. The MALDI counterpart from two additional brains is shown in Fig. 10. Data in **b** are shown for four biological replicates as mean ± SD. Source data are provided as a Source Data file.

In addition to palmitate, we extended our flux imaging approach to stearate, an important precursor for very long chain fatty acids in brain and the most abundant fatty acid in brain phospholipids. Indeed, we found multiple very long chain fatty acids to be altered in our untargeted metabolomics comparison of ipsilateral and contralateral brain tissue (Fig. 2). Similar to de novo synthesis of palmitate from acetyl-CoA, stearate is synthesized via elongation of palmitate through a series of enzymatic reactions (Fig. 9a). In contrast to palmitate synthesis in the cytosol, fatty acid elongation happens in the endoplasmic reticulum (ER)^54^. Notwithstanding, LC/MS measurement of bulk brain tissue containing glioma show a proportion of stearate in which all of its carbons are labeled, suggesting that the pool of acetyl-CoA used to elongate palmitate is enriched with ^13^C (Fig. 9b).

**Fig. 10:**
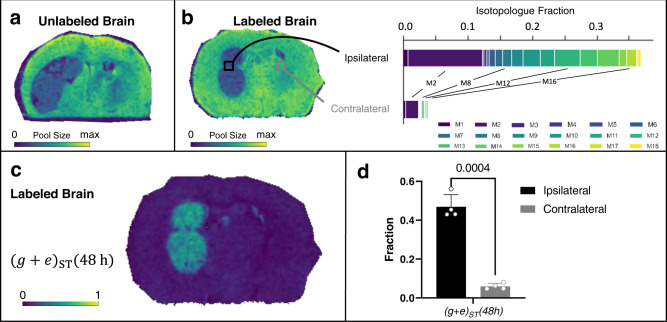
Stearate isotopologue imaging with MALDI. **a** MALDI imaging shows stearate pool size in the unlabeled tumor brain. **b** DESI imaging of the labeled brain indicates stearate pool size, with two regions of interest (ROI) selected for comparison of stearate labeling patterns. The tumor (ipsilateral) region shows higher labeling than the non-tumor (contralateral) region. **c** Flux image after applying SISA to a labeled tumor brain shows that the distribution of stearate turnover, *(g* + *e)*_*ST*_*(48* *h)*, is higher in the tumor region. **d** Comparison of *(g* + *e)*_*ST*_*(48* *h)* in the tumor site versus non-tumor tissue in four biological replicates. Data shown as mean ± SD. *p* value from paired *t* test (two-tailed). Stearate flux images for all four biological replicates with corresponding microscopy images are shown in Supplementary Fig. 11. The DESI counterpart from two additional brains is shown in Fig. 9. Source data are provided as a Source Data file.

As we did for palmitate, we used DESI and MALDI MSI to measure stearate from the brains of unlabeled and labeled mice (Figs. 9–10). Data from IMS and unlabeled samples did not reveal any interferences (Supplementary Figs. 3d, 14, and  15), thereby allowing us to use all of the stearate isotopologues in our analysis. Similar to MSI data from palmitate, DESI and MALDI provided inconsistent results about stearate pool size (Figs. 9c, d and 10a, b and Supplementary Figs. 14 and 15). In DESI, images from both the unlabeled and labeled samples indicated increased stearate levels in glioma compared to other tissue regions. MALDI provided the opposite pattern, with stearate signal being lower in the tumor. As for palmitate, LC/MS data showed no differences in stearate pool size between the tumor and non-tumor hemispheres (see Supplementary Fig. 5). These results further underscore the challenges of comparing the levels of metabolites across regions of tissue with diverse composition. In contrast to pool-size data, the same trends in stearate isotopologue abundances were observed from DESI, MALDI, and LC/MS (see Supplementary Fig. 5)

We then extended SISA to map the fractional flux of palmitate to stearate elongation. Palmitate, generated from at most 8 tracer-derived acetyl-CoA molecules, and stearate, generated from an elongation step, are not treated identically by SISA. Specifically, elongation of unlabeled palmitate with a labeled acetyl-CoA will produce an M2 stearate molecule. As there will be a large pool of unlabeled palmitate at the beginning of the experiment, the M2 peak will be higher than that expected from complete de novo synthesis of stearate from labeled acetyl-CoA. Thus, we modified the SISA model to include an elongation term of unlabeled palmitate *e*_*ST*_*(48* *h)* in addition to the de novo synthesis term *g*_*ST*_*(48* *h)*. As both terms reflect newly synthesized stearate, the fractional biosynthetic flux is *(g* + *e)*_*ST*_ *(48* *h)*. Further details are provided in the Methods. In assessing the biosynthetic flux spatially for labeled brain, strikingly, only the tumor region shows a high *(g* + *e)*_*ST*_ *(48* *h)* from both DESI and MALDI data (Figs. 9e and Fig. 10c). Replicate data for three additional labeled mice are provided in Supplementary Figs. 10 and 11. Statistical results for the four labeled mice are provided in Figs. 9f and 10d. When using data from MALDI, stearate shows on the order of 40% fractional synthesis at the tumor, compared to ~5% in the rest of the brain.

## Discussion

When performing metabolomics, LC/MS is generally recognized as the gold-standard platform. A barrier hindering the growth of MSI is the challenge of interpreting DESI and MALDI data. Not only is quantitative assessment of relative signal intensities complicated by variable matrix effects across a tissue section, but pathway activities cannot be derived from metabolite pool sizes alone. In LC/MS, a suite of experimental approaches and informatics techniques have been established that leverage stable isotope tracers to evaluate the dynamics of metabolism quantitatively. To date, however, comparable MSI experiments have been limited by a lack of established workflows and computational resources. In this work, we extended a collection of established tools typically used for the analysis of LC/MS-based metabolomics data to be compatible with MSI data. Beyond enabling SISA, the software we developed introduces basic functions for analyzing MSI data (e.g., natural abundance correction and automated generation of fractional isotopologue images) that we anticipate will be broadly applicable to a wide range of future studies. While we only processed data from DESI and MALDI here, the software is designed for all forms of MSI data that can be converted to the open imzML format, and we expect the use of multiple imaging modalities to provide complementary information. The software is freely available and can be found on GitHub (https://github.com/e-stan/imaging).

Whether an experiment uses LC/MS or MSI to measure isotopically labeled samples, it is critical that individual isotopologues be resolved for accurate interpretation of metabolic dynamics. Although isotopologue interferences can occur in LC/MS^55^, they tend to be less problematic because chromatographic separation limits the number of metabolites that appear in the same mass spectrum. In MSI, on the other hand, all of the metabolites and lipids in a specific region of tissue are simultaneously ionized. This leads to thousands of peaks in the resulting mass spectrum. The probability of having overlapping *m/z* signals therefore increases considerably, particularly when samples are isotopically labeled (Supplementary Fig. 16). In this work, we applied two strategies to identify isotopologue interferences. First, we used IMS to validate that all of the isotopologues for a given compound co-migrated. Second, we processed unlabeled samples and labeled samples in parallel to ensure that no ions in the unlabeled samples had *m/z* values overlapping with an isotopologue in the labeled samples. Even when using an instrument with over 43,000 mass resolving power at *m/z* = 554, we found a striking number of isotopologue interferences, underscoring the importance of IMS and unlabeled samples for filtering. We note that some isotopologue interferences only occurred in specific regions of tissue, such as the tumor. When not excluded, they led to spatially interesting but biologically misleading labeling patterns across the tissue. Given these challenges, MSI datasets from isotopically labeled tissues are best evaluated by targeted analysis and require validation of results to avoid erroneous conclusions.

In theory, MSI experiments performed on isotopically labeled samples do not forfeit any information content that would be obtained from an unlabeled experiment, namely metabolite pool sizes. To approximate pool size from labeled samples, the intensities of all isotopologues for a given target compound can be summed to collapse the data into a pseudo-unlabeled format. We found such an approach to be generally effective, except for metabolites with low abundance where summing the signals of multiple low-intensity isotopologues propagated measurement error. Additionally, for metabolites of low abundance or metabolites with rapid turnover, the time required to isolate tissues from animals may cause disruption of metabolite pool sizes before quenching metabolism. In our study, brains were harvested and immediately transferred to dry ice. Although such a protocol is commonly used in metabolomics, it is slower to quench metabolism than other methods such as immersion freezing, funnel freezing, freeze blowing, and head-focused microwave fixation^56^. Some reports have recommended quenching metabolism at harvest with the aforementioned techniques to minimize post-mortem changes in metabolite levels^57^. Notwithstanding, a strength of the approach that we applied to analyze isotope labeling data is that it does not rely upon pool-size information, which is historically difficult to quantitate by MSI. We provide two examples where, due to variable matrix effects, DESI and MALDI indicate opposite trends with respect to whether the compound is up or down in the tumor compared to healthy tissue.

A benefit of using MSI to study cancer metabolism is that biochemical activities within a tumor and surrounding tissues can be mapped spatially in situ. The approach presents an exciting opportunity to determine what effects local changes in environment (e.g., due to differences in nutrient availability, interactions with healthy tissues, etc.) have on the metabolism of discrete regions of the tumor. In this work, we implanted GL261 cells in the brains of mice to study a preclinical model of glioblastoma. Previous work has established that brain tumors formed from GL261 cells exhibit functional and histologic heterogeneity^27–29,58,59^. The metabolic phenotypes we examined here, however, showed a notable degree of homogeneity at the spatial resolution profiled.

An attractive feature of SISA is that it allows for quantitative analysis of lipid metabolism from brain tissue in vivo. As cancer cells within glioblastoma proliferate, they require lipids to form new membranes^60^. They can either obtain lipids by taking them up from their extracellular environment or by synthesizing them de novo. Several reports have provided support for the former, showing that multiple types of cancer cells avidly consume lipids in vitro and in vivo^61–64^. A recent study, on the other hand, concluded that breast cancer cells rely on fatty acid synthesis when they metastasize to the brain^65^. Here, we wished to determine the origin of lipids in a widely used glioblastoma model using immunocompetent mice. The GL261 cells we implanted harbored an R132H mutation in IDH1, which causes mutant enzyme to produce the oncometabolite 2-hydroxyglutarate (2HG). Synthesis of 2HG competes with de novo lipogenesis for NADPH, thereby making it metabolically advantageous for mutant IDH1 cells to take up lipids rather than synthesize them^66^. Yet, despite the potential fitness cost, we found that the gliomas synthesized approximately half of their palmitate de novo in 48 hours. For comparison, healthy tissue only synthesized ~20% of their palmitate over the same time scale. Had we only used LC/MS to analyze dissected tumors in bulk, we could not rule out the possibility that only portions of the tumor were synthesizing palmitate over the 48 hours sampled, but MSI data indicate that is not the case. DESI and MALDI experiments reveal that tumor cells within most pixels of glioma are synthesizing palmitate at relatively similar rates, which is nearly three times faster than healthy brain tissue. These findings are consistent with genetic evidence from previous studies of glioblastoma (Supplementary Fig. 17)^67^. Interestingly, compared to de novo lipogenesis flux, SISA revealed an even greater difference in palmitate elongation flux, which was approximately eight times higher in tumor tissue compared to healthy brain regions. This result is consistent with there being a greater demand for eighteen carbon acyl chains in proliferating cancer cells compared to sixteen carbon acyl chains due to their higher prevalence in membrane phospholipids^68^. It also suggests that, while fatty acid synthesis was demonstrated to be a potentially attractive therapeutic target in metastatic brain cancer^65^, fatty acid elongase might be more selective in glioblastoma.

## Methods

### In vivo ^13^C labeling experiments

Animal experiments were approved by the Institutional Animal Care and Use Committee at Washington University (assurance number A338101, protocol 19-0930 and 22-0304) and were performed in accordance with the recommendations in the Guide for the Care and Use of Laboratory Animals of the NIH^69^. Female mice (C57BL/6J, 8 weeks old) were obtained from Jackson Laboratory. Sex was not considered in this study as our primary focus was comparing different tissue regions within the same brain. Mice were housed with an ambient temperature of 22 °C, a relative humidity of 55%, and a 12-h light/dark cycle. The murine glioma cell line GL261 expressing a luciferase reporter and RFP^70^ was transduced with IDH1 R132H (pLV-R132H-YFP). The cells were grown in high-glucose DMEM with 10% fetal bovine serum and 1% penicillin/streptomycin. Cells were harvested by using trypsin, centrifuged, and resuspended in PBS (66,666 cells µL^−1^). Mice were anesthetized and 200,000 cells (3 µL) were injected into the frontal lobe of the left hemisphere of mouse brains by using a stereotaxic apparatus (2 mm lateral to the bregma and 3 mm deep at a speed of 0.6 µL min^−1^)^71^. Mice were weighed daily, and tumor burden was assessed by bioluminescence imaging (see below) as well as clinical symptoms such as neurological deficits and weight loss. The humane endpoint of the experiment was defined by loss of 20% body weight and neurological deficits. These limits were not exceeded, and all of the mice consumed the diet provided to them.

To monitor tumor growth, mice were anesthetized with isoflurane, injected intraperitoneally with luciferin (150 mg kg^−1^, Gold Bio), and subjected to bioluminescent imaging by using an IVIS Lumina Series III system (Caliper LifeSciences, PerkinElmer). After 8 days, tumors reached the desired size without impacting the mice’s behavior. Mice were then fed a liquid diet containing unlabeled glucose or U-^13^C-labeled glucose (Cambridge Isotope Laboratories) ad libitum for 48 h^33^. The diet was prepared in 50 mL tubes to allow for proper vortexing. For two mice per day, 3.06 g base mix (Teklad Custom Diet, TD.150344, Envigo) was thoroughly mixed with 4.95 g unlabeled or 5.12 g U-^13^C-labeled glucose. Next 22 mL water was added, and the tube was thoroughly vortexed. After 20 minutes, the tubes were vortexed again before transferring the diet to a liquid diet feeding tube (Bio-Serv). We would like to note that, even after optimization of the water content, the consistency made it challenging to fill the tube to a higher level, thus one tube was used per two mice.

After 48 h, mice were anesthetized with isoflurane and blood was collected by cardiac puncture. After clotting at room temperature for 30–60 min, serum was obtained by centrifugation (1000 × *g*, 10 min, room temperature). Brains were harvested and immediately fixed in carboxymethylcellulose on dry-ice followed by storage at −80 °C. Five tumor-bearing mice were prepared with unlabeled diet and four with labeled diet. The data shown in the main and supplementary figures provide a representative example of an unlabeled mouse and four labeled mice. The other unlabeled mice were used for LC/MS-based untargeted metabolomics analysis.

### Preparation of brain samples and fluorescence microscopy

Brains were embedded in 5% wt. carboxymethyl cellulose (Millipore Sigma) in water and stored at −80 °C. For DESI, 20 µm thick sections were collected at −20 °C by using a CM1860 cryostat (Leica Biosystems). Superfrost Plus slides (Thermo Fisher Scientific) were used after cleaning with ethanol. For MALDI, 10 µm thick sections were collected on SiO_2_ passivated, indium tin oxide coated polished float glass slides (Delta Technologies Limited, Loveland, CO, USA). Sections were dried under vacuum, stored at −80 °C until use, and thawed under vacuum immediately prior to analysis. Serial 50 µm thick sections were collected for extraction and LC/MS analysis after separating tumor-containing and non-tumor hemispheres (as described below).

Selected 10 µm thick tissue sections were mounted on Superfrost Plus slides (Thermo Fisher Scientific) in Fluoroshield mounting medium with DAPI (aqueous, Abcam, USA) and used for fluorescence microscopy to verify tumor location. A Leica DMi8 Thunder Imager was used to obtain images. For RFP, excitation was set to 540–580, DC was set to 585, emission was set to 592–668, and exposure time was set to 1.3 s. For DAPI, excitation was set to 375–435, DC was set to 455, emission was set to 450–490, and exposure time 59 ms.

### Standards and chemicals

LC/MS-grade acetonitrile, methanol, water, and metabolite standards were purchased from Millipore Sigma or Thermo Fisher Scientific. Ammonium bicarbonate, ammonium hydroxide, formic acid, and methylenediphosphonic (medronic) acid were purchased from Millipore Sigma and used as eluent additives for LC/MS.

### Desorption electrospray ionization mass spectrometry imaging

Samples were analyzed by using a Synapt XS with a DESI source (Waters Corporation). Images were collected in negative polarity by using “High Resolution Mode” (FWHM resolution of 43,000 for *m*/*z* = 554) with a capillary voltage of 4.0 kV and source temperature of 120 °C for mass range 70-560 *m/z*. Images were acquired by using a 50 µm × 50 µm pixel size and a 10 µm s^−1^ raster rate. The incident spray angle was 75°, and the collection angle to the mass spectrometer inlet was −7°. The DESI solvent was 98:2 methanol:water with 0.01% formic acid and 200 pg µL^−1^ leucine-enkephalin lockmass compound (Waters Corporation) infused at 3 µL min^−1^. Typically, the solvent for DESI systems is delivered via a syringe pump. However, we found that using an LC system (Waters Acquity) with a 1:100 splitter assembly (Agilent Technologies) allows for delivering a more constant flow and eliminates the need to refill the syringe between samples.

### Traveling wave ion mobility spectrometry

DESI samples were analyzed by using the same source conditions as imaging (except for extending the mass range to 50-1200 *m/z*), and each measurement integrated several manually selected regions on each tissue section. For direct infusion of standards, samples were analyzed by using the ESI source in negative polarity in “Resolution Mode” with a capillary voltage of 2.2 kV and a source temperature of 140 °C over a mass range of 50–1200 *m/z* on the Synapt XS. The direct infusion solvent was 98:2 methanol:water with 0.01% formic acid and 200 pg µL^−1^ leucine-enkephalin lockmass compound at 10 µL min^−1^. TWIMS settings using 25.0 V wave height and a wave velocity linear ramp from 1000 m s^−1^ to 300 m s^−1^ over 100% of the cycle were applied.

### Matrix-assisted laser desorption ionization mass spectrometry imaging

Before MALDI analysis, matrix coating (either 12 or 14 passes) was applied to the desiccated tissue sections by using an HTX M5 sprayer (HTX Technologies) with 10 mg mL^−1^ *N*-(1-Naphthyl) ethylenediamine dihydrochloride (NEDC; Sigma), dissolved in 70:30 methanol:water. The spraying parameters were set as follows: 80 °C nozzle temperature, 0.1 mL min^−1^ flow rate, 1000 mm min^−1^ velocity, 2 mm track spacing, 10 psi pressure, 3 L min^−1^ gas flow rate, and 10 s drying time for each pass.

MALDI samples were analyzed by using a timsTOF fleX with a MALDI source (Bruker Daltonics). All MALDI images were collected in negative polarity in MS1 mode at 50 μm pixel size. The laser was set as a single focused beam with a laser power of 85%, a laser frequency of 10 kHz, and 200 shots per pixel. The mass range was 50-400 *m/z*. Mass calibrations were performed by using 1 mM sodium formate solution. AMP, stearate, and palmitate were analyzed from sections with 12 layers of matrix coating, while NAA was analyzed in sections with 14 layers applied.

### LC/MS sample preparation

Sections of 50 µm brain tissue were cut into tumor and non-tumor hemispheres with a razor blade and collected into Eppendorf tubes while being kept frozen in the cryostat. The samples were then extracted with 2:2:1 acetonitrile, methanol, water at a ratio of 80 µL per mg wet weight. The weight was calculated based on area and thickness of the sections. The solvent was added to the tissue and vigorously vortexed. For serum analysis, 5 µL serum was mixed with 45 µL of a 4:4:1 mixture of acetonitrile, methanol, water, and vortexed. Extraction blanks were prepared with 5 µL water instead of serum. All samples were kept at −20 °C overnight. After centrifugation at 14,000 × *g* for 10 min at 4 °C, the supernatant was transferred to an LC/MS vial.

### Liquid chromatography

Metabolites were separated via hydrophilic interaction liquid chromatography (HILIC) by using a SeQuant ZIC-pHILIC column (100 × 2.1 mm, 5 µm, polymer, Merck-Millipore) with a ZIC-pHILIC guard column (20 mm × 2.1 mm, 5 µm). The column compartment temperature was 40 °C and the flow rate was set to 250 µL min^−1^. The mobile phases consisted of A: 95% water, 5% acetonitrile, 20 mM ammonium bicarbonate, 0.1% ammonium hydroxide solution (25% ammonia in water), 5 µM medronic acid; and B: 95% acetonitrile, 5% water. The following linear gradient was applied: 0 to 1 min, 90% B; 12 min, 35% B; 12.5 to 14.5 min, 25% B; 15 min, 90% B followed by a re-equilibration phase of 4 min at 400 µL min^−1^ and 2 min at 250 µL min^−1^. The samples were kept at 6 °C in the autosampler. The injection volume was 5 µL.

### High-resolution mass spectrometry

A Vanquish UHPLC system was coupled to an Orbitrap ID-X Tribrid mass spectrometer (Thermo Fisher Scientific) via electrospray ionization with the following source conditions: sheath gas flow 50 arbitrary units (Arb), auxiliary gas flow 10 Arb, sweep gas flow 1 Arb, ion transfer tube temperature 300 °C, and vaporizer temperature 200 °C. The RF lens value was 60%. Data were acquired in negative and positive polarity with a spray voltage of 2.8 kV and 3.5 kV, respectively. MS1 data were acquired from 67 to 900 *m/z* at a resolution of 120,000 with an automatic gain control (AGC) target of 2e5 and a maximum injection time of 200 ms in polarity switching mode. MS2 data for metabolite identification were acquired at a resolution of 15,000 with an AGC target of 2.5e4 and a maximum injection time of 70 ms in negative and positive mode separately. A 5 ppm mass error and 10 s dynamic exclusion were applied.

### LC/MS-based untargeted metabolomics data processing

LC/MS data from tumor and non-tumor hemispheres of unlabeled mouse brains were processed with Compound Discoverer 3.3. Features were grouped (isotopes and adducts) and background compounds (ratio sample to blank ≥5) were removed. Peak areas were normalized by constant median. Compounds were then filtered based on a CV of <20% in at least one of the two sample groups, resulting in a total of 459 compounds, which were used to generate the PCA plot (Fig. 2a). A *p* value threshold of 0.05 resulted in 166 compounds and was used to generate the heatmap (Fig. 2b). Metabolite identifications used in Fig. 2c, d were obtained by matching MS/MS spectra to mzCloud and had a matching score > 70.

### IMS data processing

To determine CCS values, extracted ion mobilograms corresponding to target metabolites were extracted from the raw data generated from the DESI system. Peak smoothing and CCS calculations were performed in Python, and the code for performing this analysis is available on GitHub (https://github.com/e-stan/imaging). CCS values were compared to those published in the Unified CCS Compendium^72^. Ions whose extracted ion mobilograms showed multiple or distorted peaks were excluded from use as MSI images.

### MSI data processing

DESI imaging data were first opened through MassLynx software (Waters Corporation) to evaluate spectra quality and perform a lockmass correction. The raw data were then converted to imzML by using HDI software (Waters Corporation). MALDI data were first processed in the SCiLS software (Bruker Corporation) and exported to imzML. The converted data from unlabeled samples were then analyzed with METASPACE^34^ to annotate the detected ions, and the converted data were subjected to a mass recalibration with adaptive pixel mass recalibration using the high-confidence METASPACE annotations (lists of all metabolites identified with an FDR < 10% are provided as Supplementary Table 1–3). Adaptive pixel mass recalibration^73^ has been integrated into the Python-based software developed in this study. Although the raw MSI data contained thousands of unique signals, only a small number of annotations were returned by METASPACE. This is a consequence of overlapping *m/*z peaks that prevent natural abundance isotope pattern matches to be made within the METASPACE software. We then further filtered the metabolite annotations returned by METASPACE to remove metabolites that do not show isotope incorporation in the labeled samples. This process resulted in a list of target metabolites for evaluation. To analyze the resulting recalibrated data, a target list of *m/z* values of interest was calculated from the molecular formulas of the target metabolites. The intensity of each of these masses was then extracted from the data and formatted into a *n* × *w* × *h* tensor where *n* is the number of *m/z* values of interest, *w* is the width of the image in pixels, and *h* is the height of the image in pixels. Then, the images were segmented into non-tissue background and tissue by performing PCA-based dimensionality reduction, followed by k-*means* clustering (*k* = 2). Images were subsequently de-noised with the application of a Gaussian blur 3 × 3 pixel filter. Tissue pixels were natural isotope abundance corrected and further processed into isotopologue fractional images or flux images with SISA. The code and scripts used to process the raw data into the images and graphs shown in this study are available on GitHub.

### SISA

To calculate the fractional flux of palmitate synthesis from a labeled tracer (glucose), a non-linear model derived from classical ISA^31^ was employed. Classical ISA (Eq. 3) models the labeling pattern of a product metabolite ($P$) containing $n$ precursor subunits as a polynomial function of tracer precursor labeling ($T$), natural abundance precursor labeling (*N*), tracer dilution ($D$), and product synthesis flux ($g(t)$).

3 $$\sum {P}_{i}=	\, g(t){\left({\left(1-D\right)}\left(N_{0}+{N}_{1}+{N}_{2}\right)+D\left({T}_{0}+{T}_{1}+{T}_{2}\right)\right)}^{n} \\ 	+\left(1-g(t)\right){\left({N}_{0}+{N}_{1}+{N}_{2}\right)}^{n}$$

The SISA approach presented in this study differs from classical ISA in that classical ISA assumes that the precursor labeling is known. This is reasonable when the tracer and precursor are the same species. However, as glucose was used as the tracer, acetyl-CoA (the precursor) labeling is unknown and cannot be measured on the MSI platforms. Thus, the presented ISA method jointly infers the precursor labeling and the fractional flux. This removes the $D$ and $T$ terms and replaces it with a precursor labeling term ($X$) that must be inferred, along with the fractional turnover of palmitate, *g(t)* (Eq. 4).

4 $$\sum {P}_{i}=\,{g}_{}\left(t\right){({X}_{0}+{X}_{1}+{X}_{2})}^{n}+\left(1-{g}_{}\left(t\right)\right){\left({N}_{0}+{N}_{1}+{N}_{2}\right)}^{n}$$

In SISA, four unknown variables (${X}_{0},\,{X}_{1},\,{X}_{2}$, and $g\left(t\right)$) must be inferred. While this is more than the two parameters that are inferred in classical ISA, the system is still overdetermined in the case of palmitate. Palmitate is made of 16 carbons (8 acetyl CoA subunits), thus when Eq. 2 is expanded and terms are grouped based on mass, there is a system of seventeen independent equations that is sufficient to infer $g\left(t\right)$ and the labeling of the precursor pool accurately. The model parameters are inferred by minimizing the sum of squared errors between observed and fit labeling (${P}_{i}$) with unconstrained numerical optimization within SciPy^74^.

To calculate the fractional flux of stearate synthesis via elongation of palmitate, we adapted an existing ISA approach^49^ that separates newly synthesized palmitate into elongation of unlabeled palmitate, ${e}_{{ST}}(t),$ and elongation of newly synthesized, labeled palmitate, ${g}_{{ST}}(t)$, Eq. 5.

5 $$\sum {P}_{i}=	\, {g}_{{ST}}\left(t\right){\left({X}_{0}+{X}_{1}+{X}_{2}\right)}^{9}+{e}_{{ST}}\left(t\right)({X}_{0}+{X}_{1}+{X}_{2}){\left({N}_{0}+{N}_{1}+{N}_{2}\right)}^{8} \\ 	+\left(1-{g}_{{ST}}\left(t\right)-{e}_{{ST}}(t)\right){\left({N}_{0}+{N}_{1}+{N}_{2}\right)}^{9}$$

To solve this system, we again rely on numerical optimization (constrained) within SciPy^74^ to find the model parameters yielding the smallest sum of squared errors between observed and fit labeling. This time, however, we infer ${g}_{{ST}}(t)$, ${e}_{{ST}}(t)$, and the precursor (${X}_{1},{X}_{2},\,{X}_{3}$) labeling.

### Metabolite identification

Authentic metabolite standards were used to identify metabolites via exact mass, retention time, MS/MS spectral matches, and CCS.

### Error propagation analysis

To assess the impact of measurement error on the SISA results, we performed an error propagation analysis by first calculating the measurement error of palmitate and stearate in an unlabeled DESI brain MSI dataset. As palmitate and stearate will not have isotopic label incorporation, the observed labeling patterns can be compared to the natural abundance pattern and the mean error across all isotopologues was used to quantitate measurement error. Next, we performed a synthetic analysis where a gradient of random, Gaussian noise was added to palmitate and stearate labeling patterns generated from the SISA models of palmitate and stearate with randomly sampled $g\left(t\right)$, $e\left(t\right)$ (for stearate only), $D$, and $T$ values. The resulting, noisy labeling patterns were then analyzed with SISA to calculate $g\left(t\right)$ and $e\left(t\right)$ values that were compared to the true values used to generate the labeling patterns. For palmitate, classical ISA was also used to infer $g\left(t\right)$. The error between calculated versus true flux parameters were then plotted against the simulated measurement error and a linear regression was computed to capture the effect of noise on the flux estimates. The fit regression was then used to project the measurement error computed in the DESI MSI dataset into expected error range for the calculated $g\left(t\right)$ and $e\left(t\right)$ values.

### Reporting summary

Further information on research design is available in the Nature Portfolio Reporting Summary linked to this article.

## Supplementary information

Supplementary Information

Reporting Summary

## Data Availability

The MALDI and DESI MSI data were uploaded to METASPACE and are available at https://metaspace2020.eu/project/MSH_MSI_SISA_2023. LC/MS data were uploaded to the Metabolomics Workbench^75^ with project identifier PR001630 [10.21228/M80T52]. The publicly available transcriptomics dataset is available at the Gene Expression Omnibus under accession number GSE147352. Source data are provided with this paper.

## Source data

Source Data

## Code availability

Source code for the analyses presented in this study can be found on GitHub (https://github.com/e-stan/imaging), which is available under the Zenodo identifier 10.5281/zenodo.7778443^76^. The GitHub site also contains links to web-based Google Colaboratory notebooks that can process raw MSI data from labeled samples without the need to install software locally.
