# Supplementary material for: Using mass spectrometry imaging to map fluxes quantitatively in the tumor ecosystem
Source: Nat Commun. 2023 May 19;14:2876. doi: 10.1038/s41467-023-38403-x (PMC10199024; doi:10.1038/s41467-023-38403-x)
Supplement: Supplementary file 1 — Supplementary Information [file 41467_2023_38403_MOESM1_ESM.pdf]

Supplementary Material

Using Mass Spectrometry Imaging to Map Fluxes Quantitatively in the Tumor Ecosystem

Michaela Schwaiger-Haber[1,2,3,6], Ethan Stancliffe[1,2,3,6], Dhanalakshmi S. Anbukumar[1,2,3], Blake Sells[1,2,3], Jia Yi[1,2,3], Kevin Cho[1,2,3], Kayla Adkins-Travis[1,2,3], Milan G. Chheda[3,4,5], Leah P. Shriver[1,2,3], and Gary J. Patti[1,2,3,5]

[1] Department of Chemistry, Washington University in St. Louis, St. Louis, MO, USA
[2] Center for Metabolomics and Isotope Tracing, Washington University in St. Louis, St. Louis, MO, USA
[3] Department of Medicine, Washington University in St. Louis, St. Louis, MO, USA
[4] Department of Neurology, Washington University in St. Louis, St. Louis, MO, USA
[5] Siteman Cancer Center, Washington University in St. Louis, St. Louis, MO, USA

[6] These authors contributed equally.

Corresponding author: Gary J. Patti (gjpattij@wustl.edu)

Table of Contents

Supplementary Figure 1. Comparison of DESI and MALDI data. 3

Supplementary Figure 2. *N*-Acetylaspartate (NAA) is synthesized by GL261 cells in vitro. 4

Supplementary Figure 3. DESI-TWIMS analysis. 5

Supplementary Figure 4. Microscopy to verify tumor sites. 6

Supplementary Figure 5. Validation of MSI isotopologue fractional imaging with LC/MS. 7

Supplementary Figure 6. Palmitate isotopologue imaging with DESI. 8

Supplementary Figure 7. Palmitate isotopologue imaging with MALDI. 9

Supplementary Figure 8. Palmitate and stearate background contamination 10

Supplementary Figure 9. SISA error analysis. 11

Supplementary Figure 10. Fluorescence microscopy to verify tumor site and DESI replicates. 12

Supplementary Figure 11. Fluorescence microscopy to verify tumor site and MALDI replicates. 13

Supplementary Figure 12. SISA validation for DESI and MALDI by using LC/MS. 14

Supplementary Figure 13. Serum data supports that fatty acid synthesis occurs in the brain. 15

Supplementary Figure 14. Stearate isotopologue imaging with DESI. 16

Supplementary Figure 15. Stearate isotopologue imaging with MALDI. 17

Supplementary Figure 16. Spectral comparison of unlabeled and labeled MSI data. 18

Supplementary Figure 17. Transcriptomics data from human samples. 19

Supplementary Table 1. Metabolites detected and identified within the DESI MSI data. 20

Supplementary Table 2. Metabolites detected and identified within the MALDI MSI data acquired with 12 matrix layers. 21

Supplementary Table 3. Metabolites detected and identified within the MALDI MSI data acquired with 14 matrix layers. 22

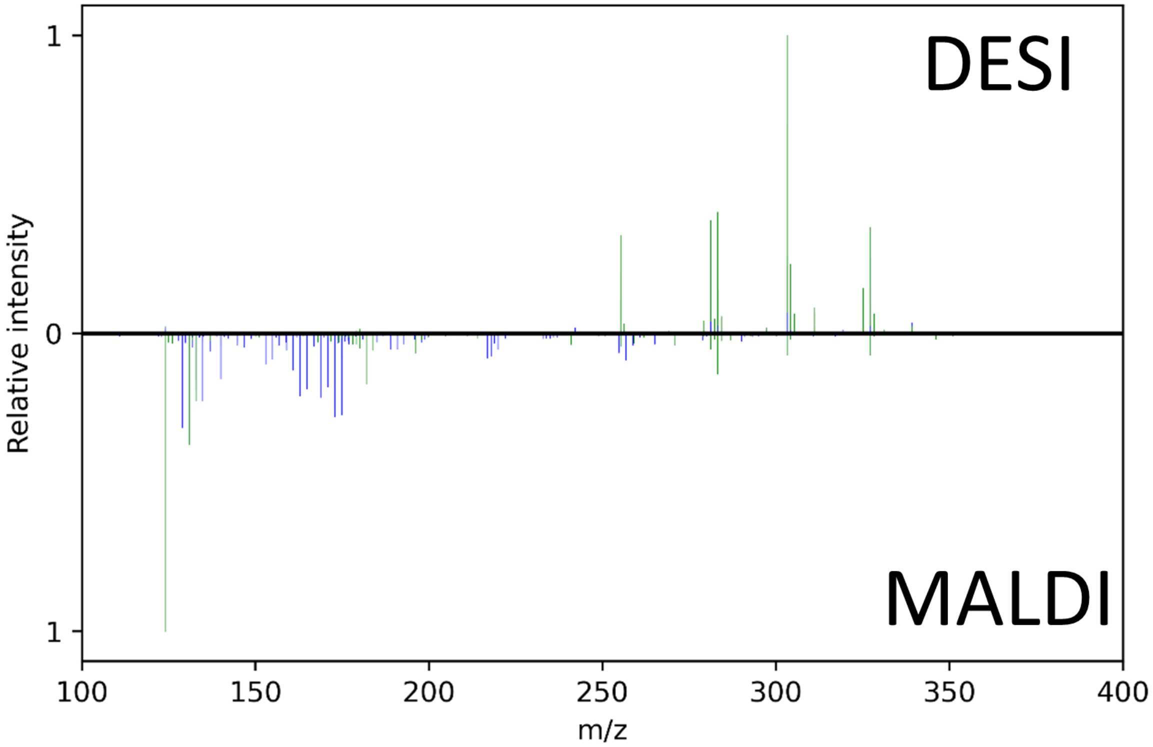

Supplementary Figure 1. Comparison of DESI and MALDI data. Mass spectra acquired with DESI (top) or MALDI (bottom) from two slices of the same mouse brain are shown. Mass spectra were averaged across the tissue region. Green peaks are shared between both DESI and MALDI spectra, while blue peaks are unique to each spectrum. There are striking differences in both sensitivity and coverage of analytes between the two techniques. The base peak in the DESI spectrum is arachidonate, which is present at a low level in the MALDI spectrum. In the MALDI spectrum, however, matrix and other analytes are uniquely detected in the low mass region, demonstrating the differences between the two techniques.

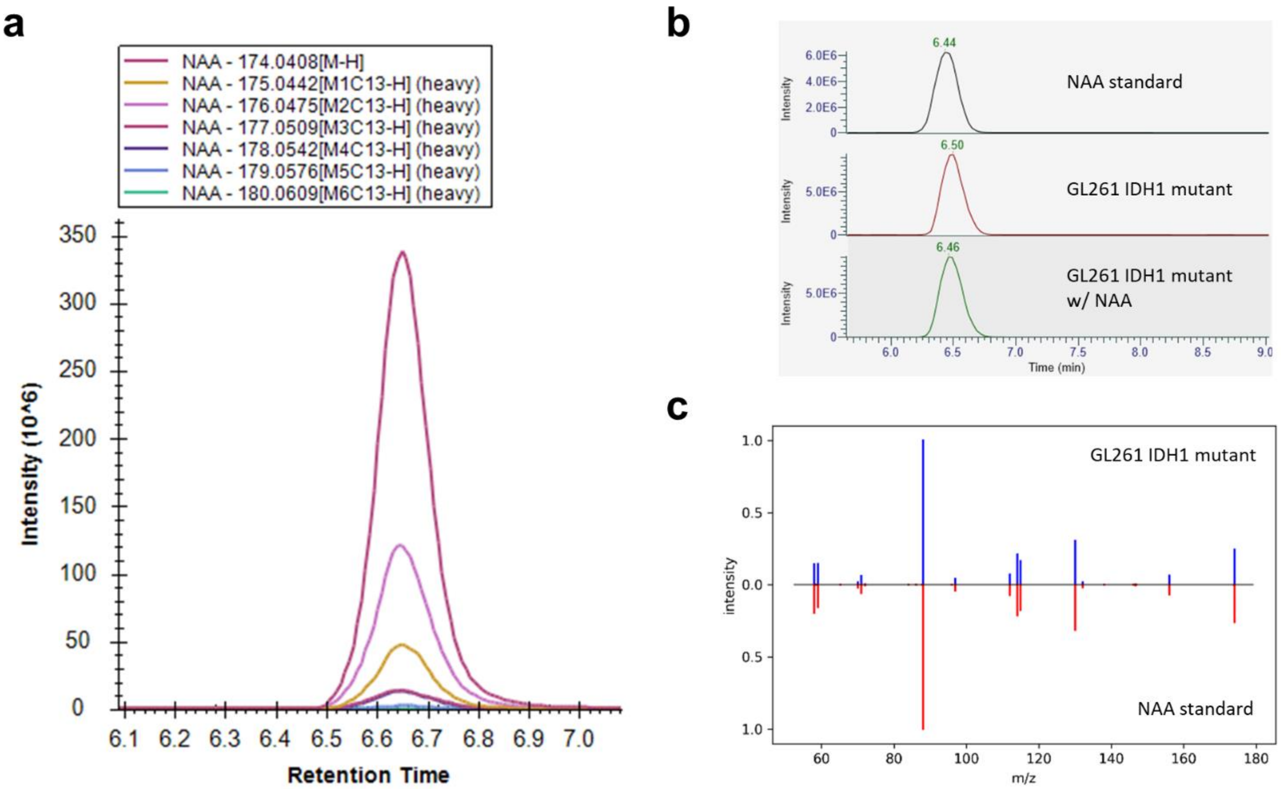

Supplementary Figure 2. *N*-Acetylaspartate (NAA) is synthesized by GL261 cells in vitro. a) Analysis of GL261 IDH1 mutant (R132H) cell extracts show incorporation of ^{13}C-labeled carbons from glucose into NAA. Cells were cultured in DMEM with 10% FBS and 1% penicillin/streptomycin and were labeled with 1,2-^{13}C glucose for 24 h. b) Chromatograms of an NAA standard (top), a GL261 cell extract (middle), and a cell extract spiked with NAA (bottom) show matching retention times. c) Mirror plot confirming matching MS/MS spectra of the GL261 cell extract (top) and an NAA standard (bottom).

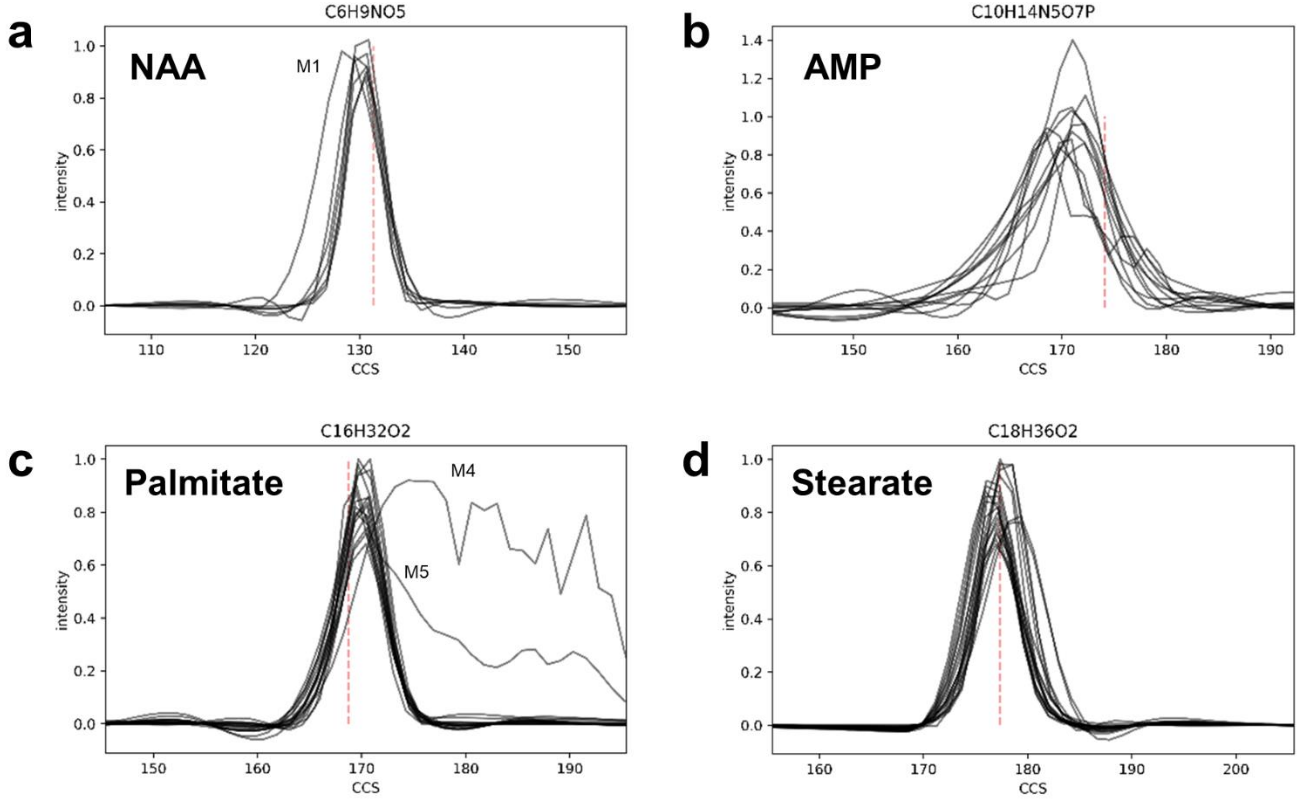

Supplementary Figure 3. DESI-TWIMS analysis. Ion mobility data for different isotopologues of a) NAA, b) AMP, c) palmitate, and d) stearate were inspected for metabolite identification and detection of interferences. For NAA (a), an interference in the M1 isotopologue was detected. For AMP (b), the signal was low, but LC/MS data confirmed the isotopologue pattern. For palmitate (c), the M4 and M5 showed additional peaks and were thus excluded from SISA analysis. Database values are shown as a red dashed line. NAA *N*-acetylaspartate, AMP adenosine monophosphate.

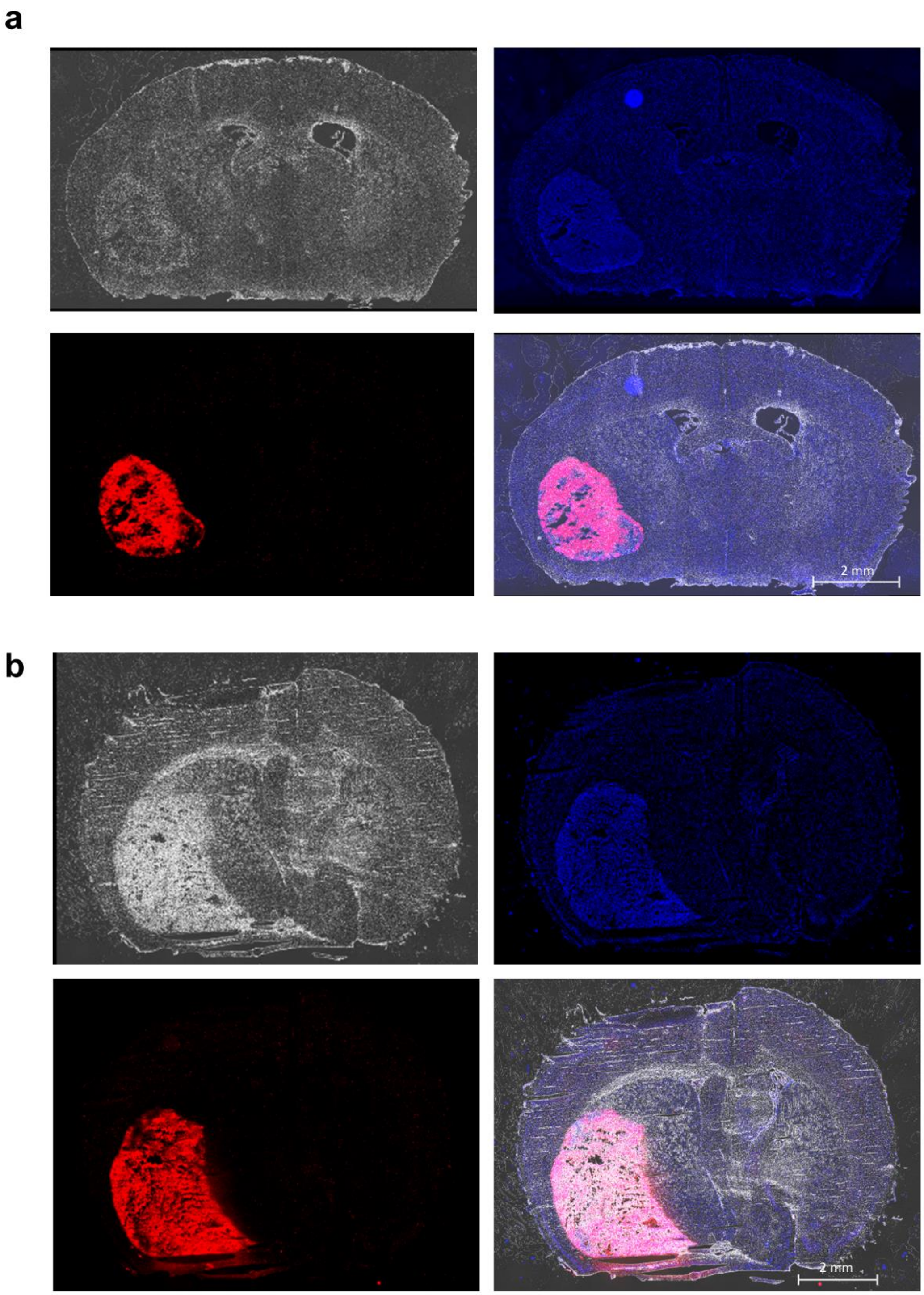

Supplementary Figure 4. Microscopy to verify tumor sites. GL261 IDH1 mutant cells were engineered to express RFP. Brightfield, DAPI, RFP, and overlay of all three signals for the unlabeled brain (a) and the labeled brain (b) that are shown in the main DESI figures. In the overlay, the tumor is indicated by pink color. All four labeled brains are shown as overlays in Supplementary Figure 10 (DESI) and Supplementary Figure 11 (MALDI). To confirm tumor location, at least three independent sections of each brain were imaged. All showed similar results.

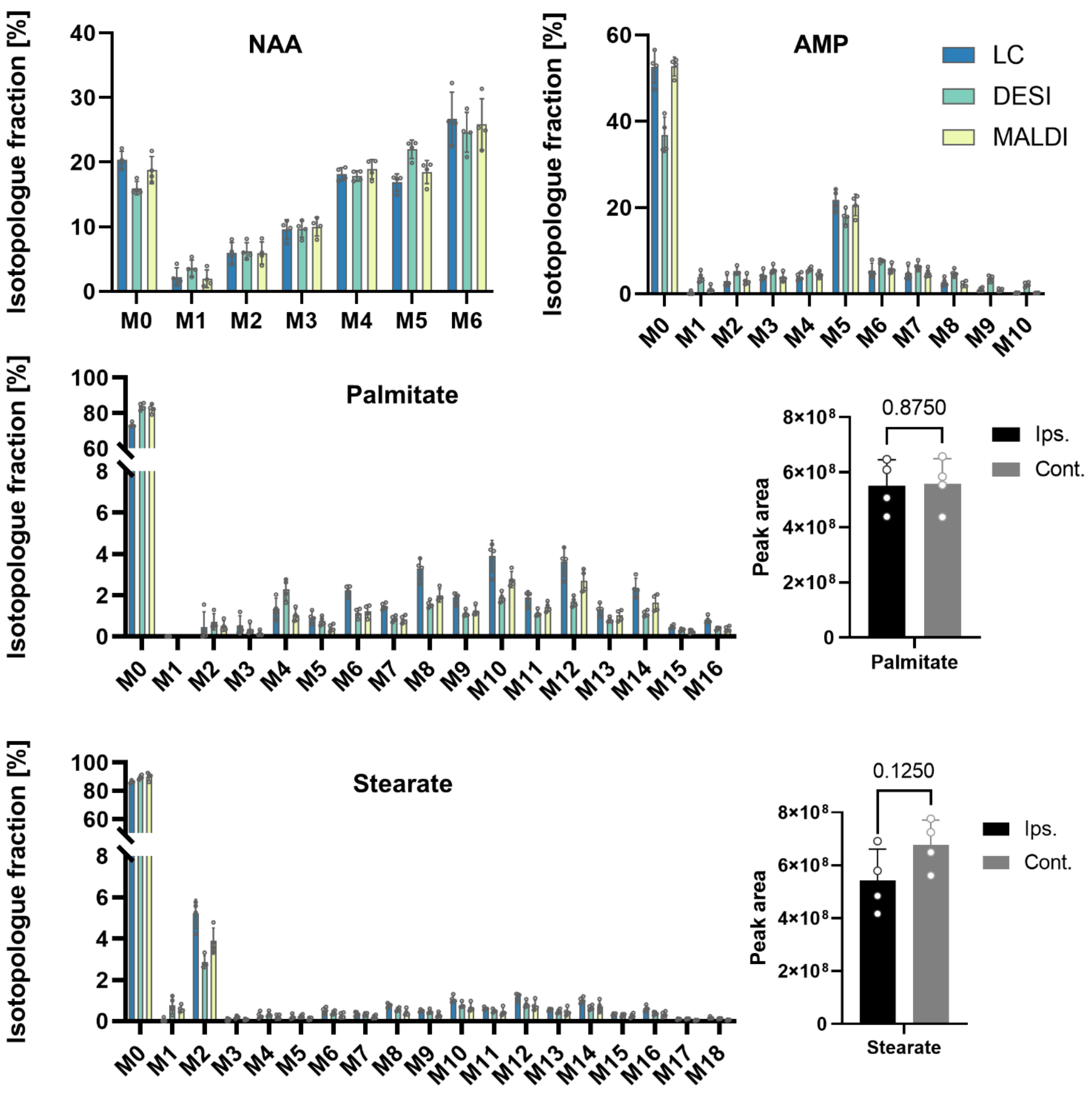

Supplementary Figure 5. Validation of MSI isotopologue fractional imaging with LC/MS. Isotopologue fractions for *N*-acetylaspartate (NAA), adenosine monophosphate (AMP), palmitate, and stearate were determined by averaging across the whole-brain samples. For palmitate, the M1 was excluded from all data due to an interference. Despite using different sample preparation and measurement approaches, DESI, MALDI, and LC/MS data show similar trends in metabolite isotopologue distributions. Pool sizes for palmitate and stearate from LC/MS data show no differences between the two hemispheres (Ips., Ipsilateral; Cont., Contralateral), paired t-test, $p > 0.05$. Data are mean ± standard deviations from four labeled mice. Source data are provided as a Source Data file.

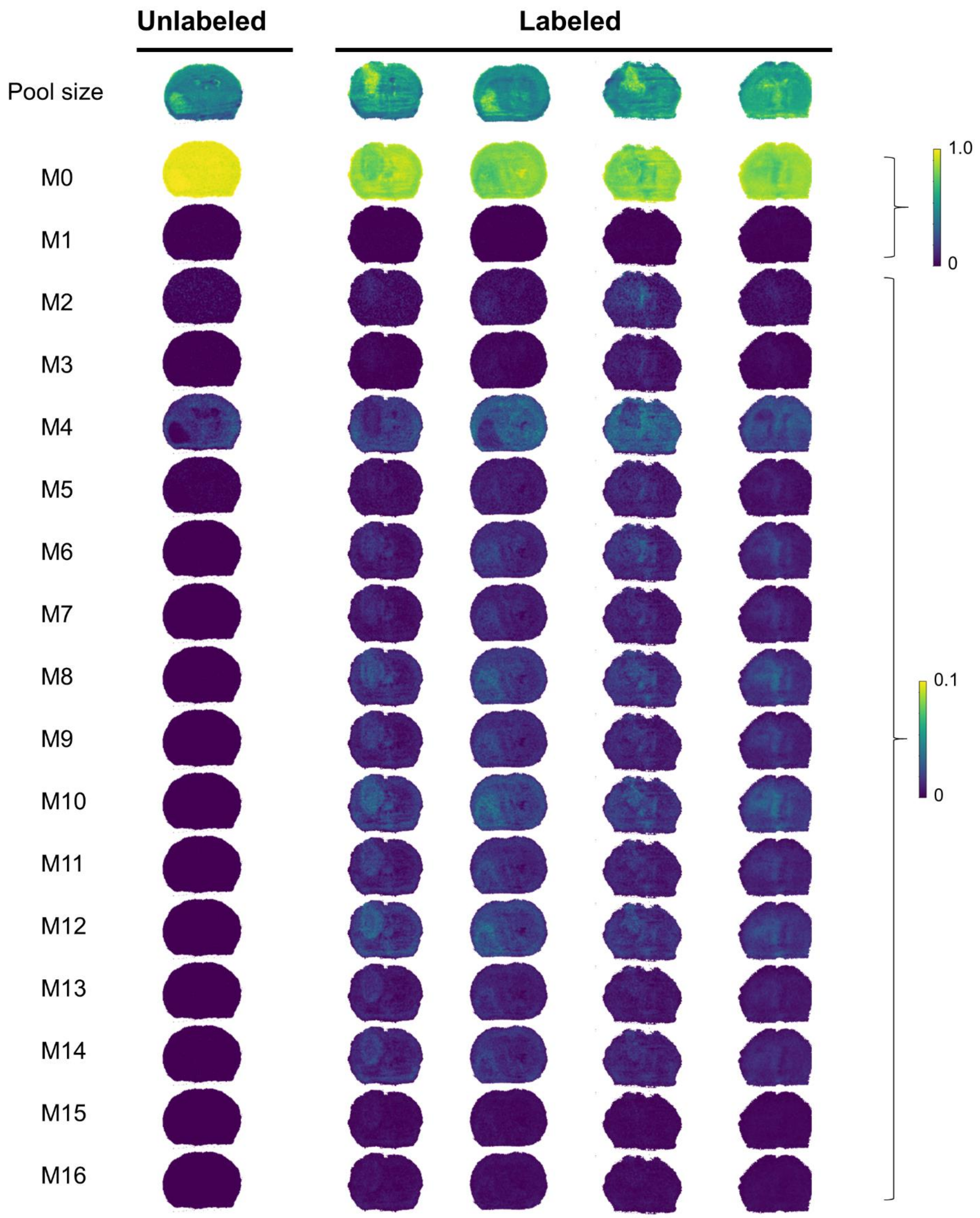

Supplementary Figure 6. Palmitate isotopologue imaging with DESI. Individual isotopologue images for palmitate in an unlabeled and four labeled tumor brains after correction for natural isotope abundance from DESI data. Note the different intensity scale for M0 and M1 compared to the other isotopologues.

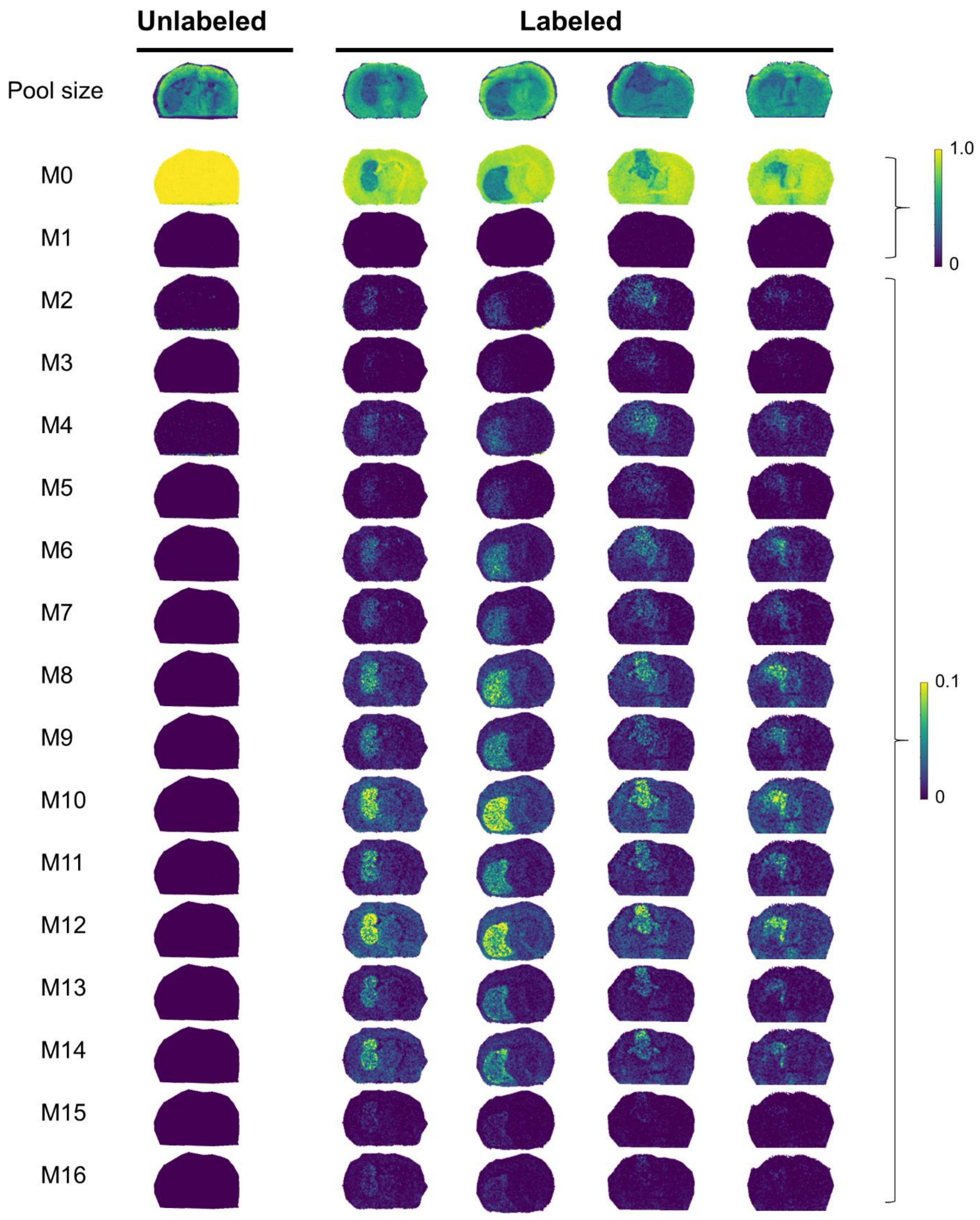

Supplementary Figure 7. Palmitate isotopologue imaging with MALDI. Individual isotopologue images for palmitate in an unlabeled and four labeled tumor brains after correction for natural isotope abundance from MALDI data. Note the different intensity scale for M0 and M1 compared to the other isotopologues.

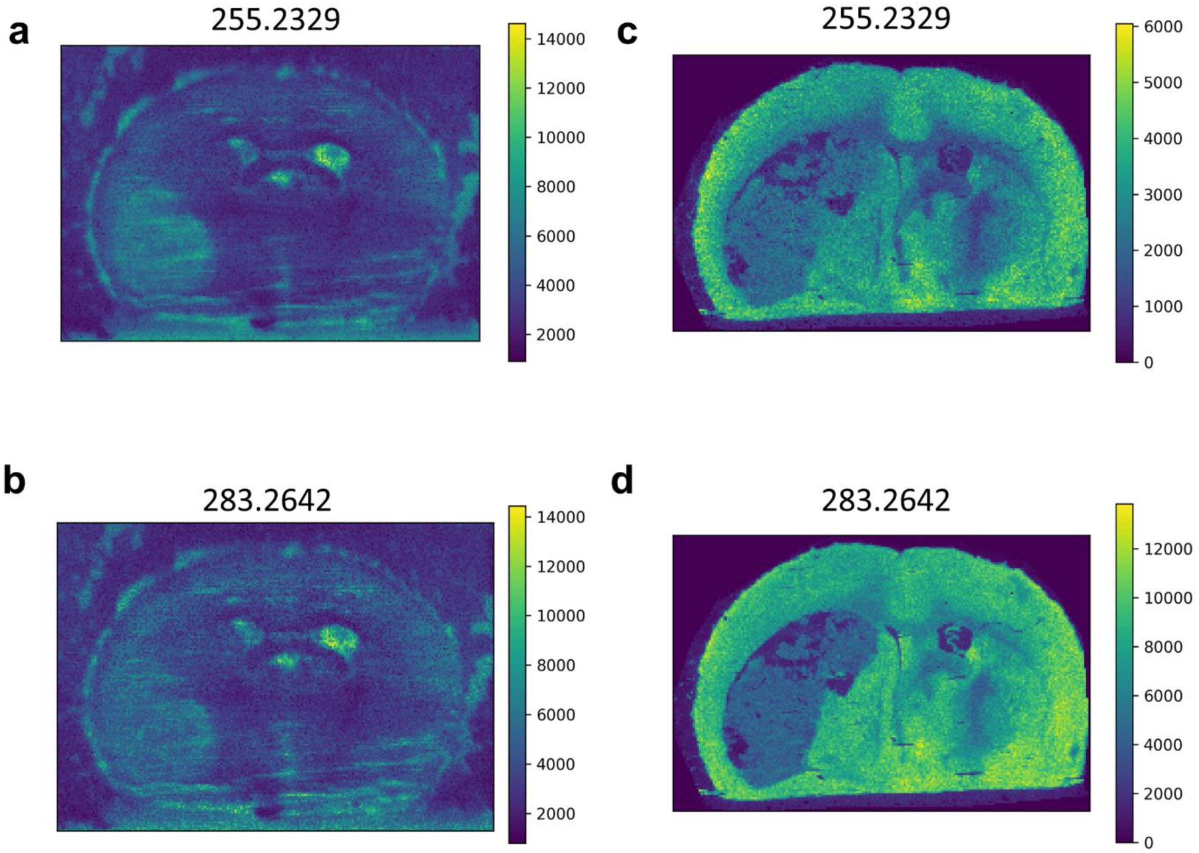

Supplementary Figure 8. Palmitate and stearate background contamination in DESI and MALDI analysis. a-b) High background signal is present for palmitate (*m/z* = 255.2329, a) and stearate (*m/z* = 283.2642, b) on the DESI slide as can be seen in the two ion images. The signal outside the brain section is comparable to the signal in the tissue region for both compounds. c-d) Low background signal is present for palmitate (c) and stearate (d) in MALDI compared to the tissue region. For the MALDI ion images, a polygonal region of interest was used to acquire the MSI data. Thus, the background signal is only the blue region that surrounds the tissue. The outer darker pixels are regions where no spectra were collected. A 15 ppm extraction window was used to generate the ion images.

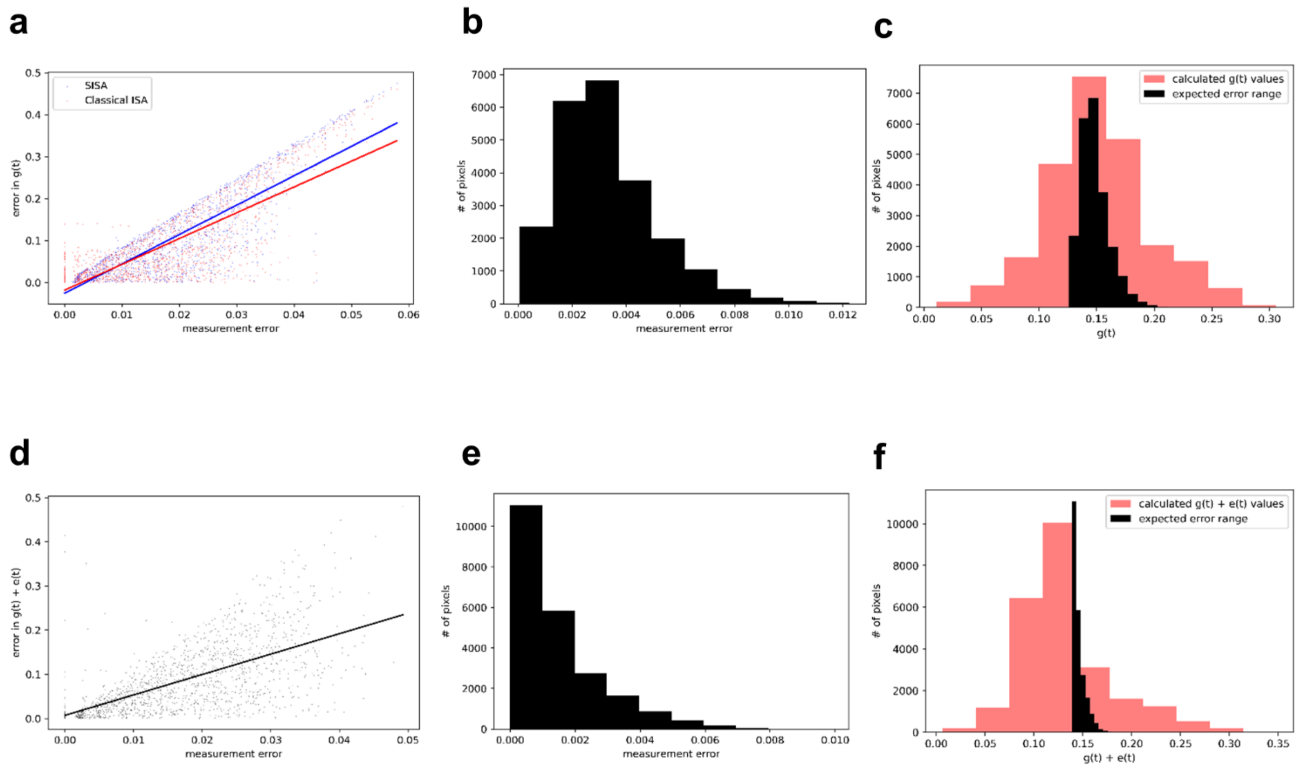

Supplementary Figure 9. SISA error analysis. Error propagation analysis for palmitate (a-c) and stearate (d-f) SISA analysis. a,d) Simulated random noise for palmitate (a) and stearate (d) was added to a theoretical labeling pattern calculated from random *g(t)*, *e(t)* (for stearate only), D, and glucose-derived acetyl-CoA labeling. The measurement error (mean difference between theoretical and “observed” labeling pattern) was calculated. SISA and classical ISA calculations were performed for palmitate, and the computed *g(t)* values were compared to the “ground-truth” values. The error in the calculation is plotted as a function of measurement error for both classical ISA and SISA workflows (a). SISA analysis was performed for stearate and the summed *g(t)* and *e(t)* values computed were compared to the true values. The error in the calculation is plotted against measurement error (d). b,e) Histograms showing the distribution of calculated measurement errors when comparing the observed labeling patterns of palmitate (b) and stearate (e) from the unlabeled DESI MSI dataset to the theoretical natural abundance labeling patterns. c,f) Measurement errors from b,e were mapped to the expected *g(t)* and *g(t)* + *e(t)* values from the regression shown in (a) and (d). The error distribution for palmitate (c) and stearate (f) is shown overlayed with the observed range of *g(t)* and *g(t)* + *e(t)* values calculated within a labeled DESI MSI dataset. The observed range of the *g(t)* and *g(t)* + *e(t)* values far exceed the range of values expected from measurement error alone.

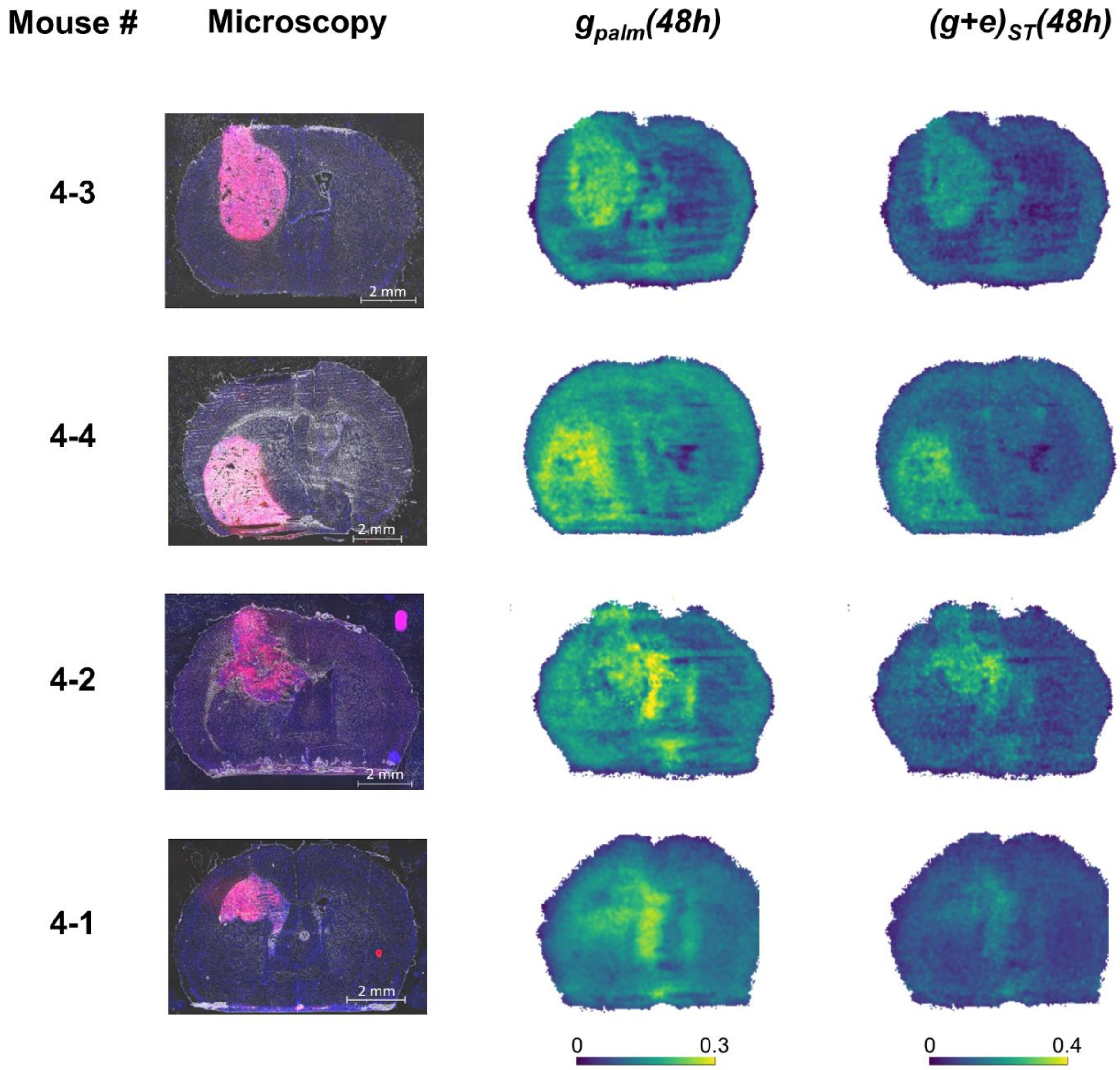

Supplementary Figure 10. Fluorescence microscopy to verify tumor site and DESI replicates. Implanted GL261 IDH1 mutant cells were engineered to express RFP. Shown are overlays of brightfield, DAPI, and RFP signals for the four labeled tumor brains. Tumors are indicated by pink color (blue + red), which overlaps with the increased palmitate and stearate turnover. To confirm tumor location by microscopy, at least three independent sections of each brain were imaged. All showed similar results. The micrographs shown here were closest to the section used for DESI MSI. $g_{palm}(48\ h)$ palmitate turnover, $(g+e)_{ST}(48\ h)$ stearate turnover.

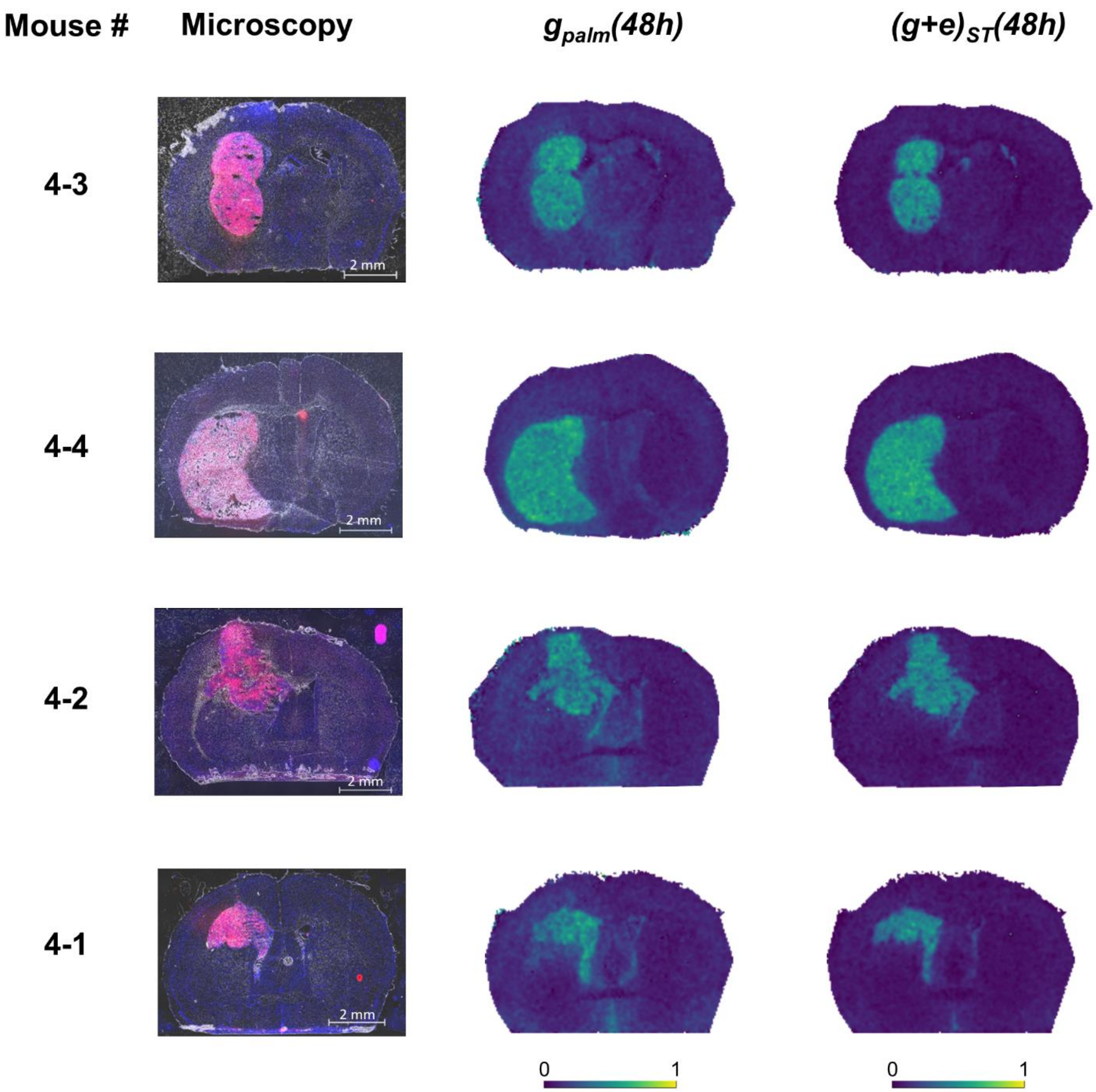

Supplementary Figure 11. Fluorescence microscopy to verify tumor site and MALDI replicates. Implanted GL261 IDH1 mutant cells were engineered to express RFP. Shown are overlays of brightfield, DAPI, and RFP signals for the four labeled tumor brains. Tumors are indicated by pink color (blue + red), which overlaps with the increased palmitate and stearate turnover. To confirm tumor location by microscopy, at least three independent sections of each brain were imaged. All showed similar results. The micrographs shown here were closest to the section used for MALDI MSI. $g_{palm}(48\ h)$ palmitate turnover, $(g{+}e)_{ST}(48\ h)$ stearate turnover.

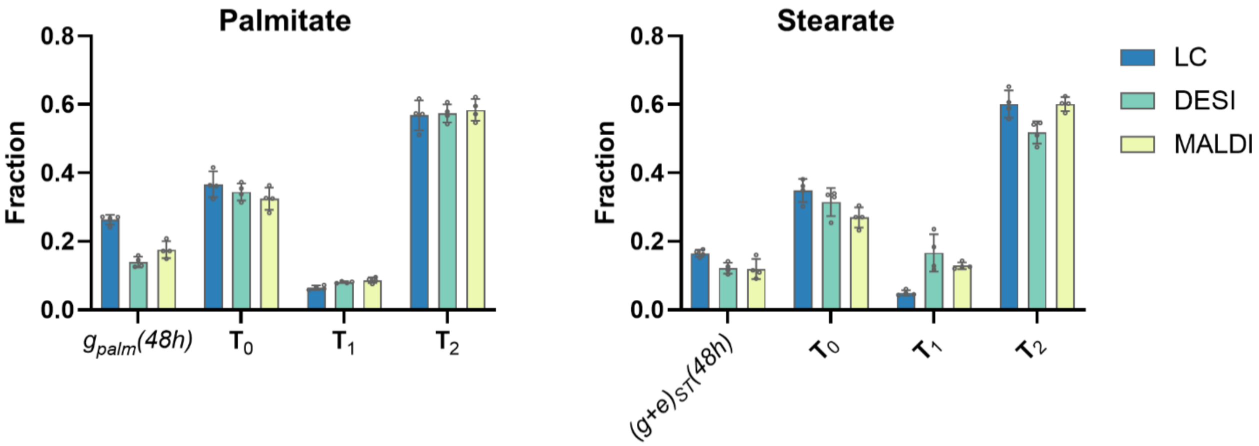

Supplementary Figure 12. SISA validation for DESI and MALDI by using LC/MS. The ISA workflow was performed by using LC/MS data from extracted tissue sections and was compared to the results obtained from DESI and MALDI MSI data by averaging across the whole brain. ISA-inferred isotopologue fractions for M0, M1, and M2 acetyl-CoA (T_0, T_1 , and T_2) show similar values with all three methods, with a minor increase in T_1 for the MALDI palmitate data and decrease in T_1 for the LC/MS stearate data. The M2 acetyl-CoA fractions (T_2) all agree within 10%. Fatty acid fractional de novo synthesis for palmitate, $g_{palm}(48h)$ is lower in the DESI and MALDI data and fractional synthesis via elongation for stearate, $(g+e)_{ST}(48\ h)$ are similar between the three methods. Of note, data are obtained from different sections from the same brains. Also, ^{12}C fatty acid contamination is a known issue that can affect different methods and provides a plausible explanation for decreasing the apparent fraction of ^{13}C-enriched fatty acid to lower *g(t)* values (see Supplementary Figure 8). Comparisons within a brain sample are not affected by such contamination and thus preferable. Data are mean ± standard deviations from four mice. Source data are provided as a Source Data file.

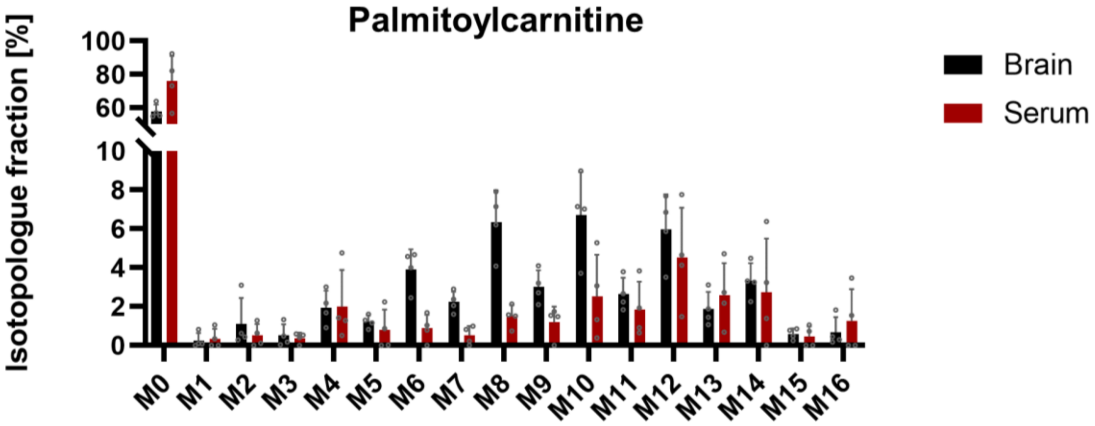

Supplementary Figure 13. Serum data supports that fatty acid synthesis occurs in the brain. Differences in palmitate labeling patterns in the brain and the serum indicate that fatty acid synthesis is occurring in the brain rather than the brain taking up labeled palmitate from the circulation. Data are shown for palmitoylcarnitine to avoid any difference in background contamination with unlabeled palmitate from materials used during sample preparation. Data are mean ± standard deviations from four mice. No labeling is observed in isotopologues above M16. Source data are provided as a Source Data file.

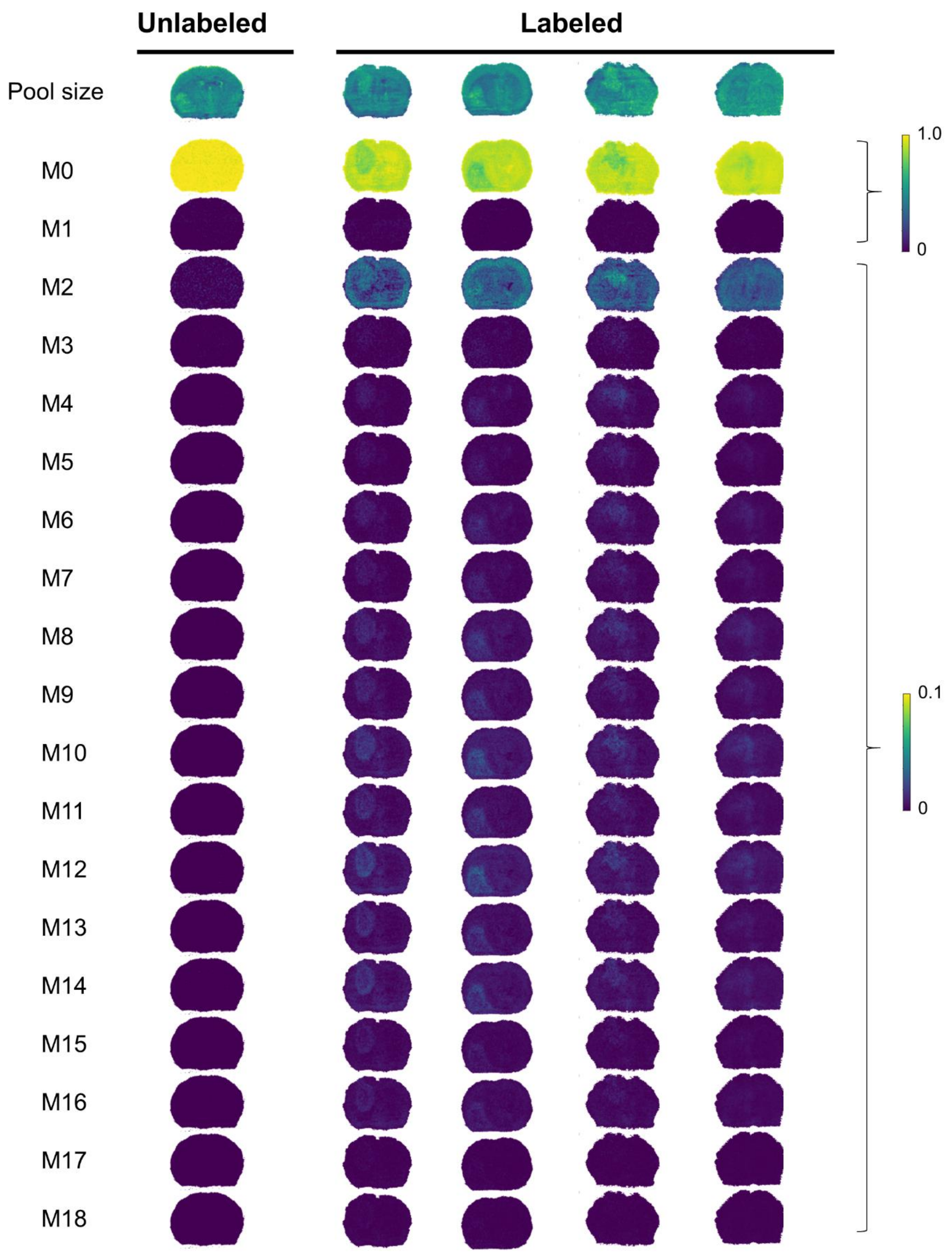

Supplementary Figure 14. Stearate isotopologue imaging with DESI. Individual isotopologue images for palmitate in an unlabeled and four labeled tumor brains after correction for natural isotope abundance from DESI data. Note the different intensity scale for M0 and M1 compared to the other isotopologues.

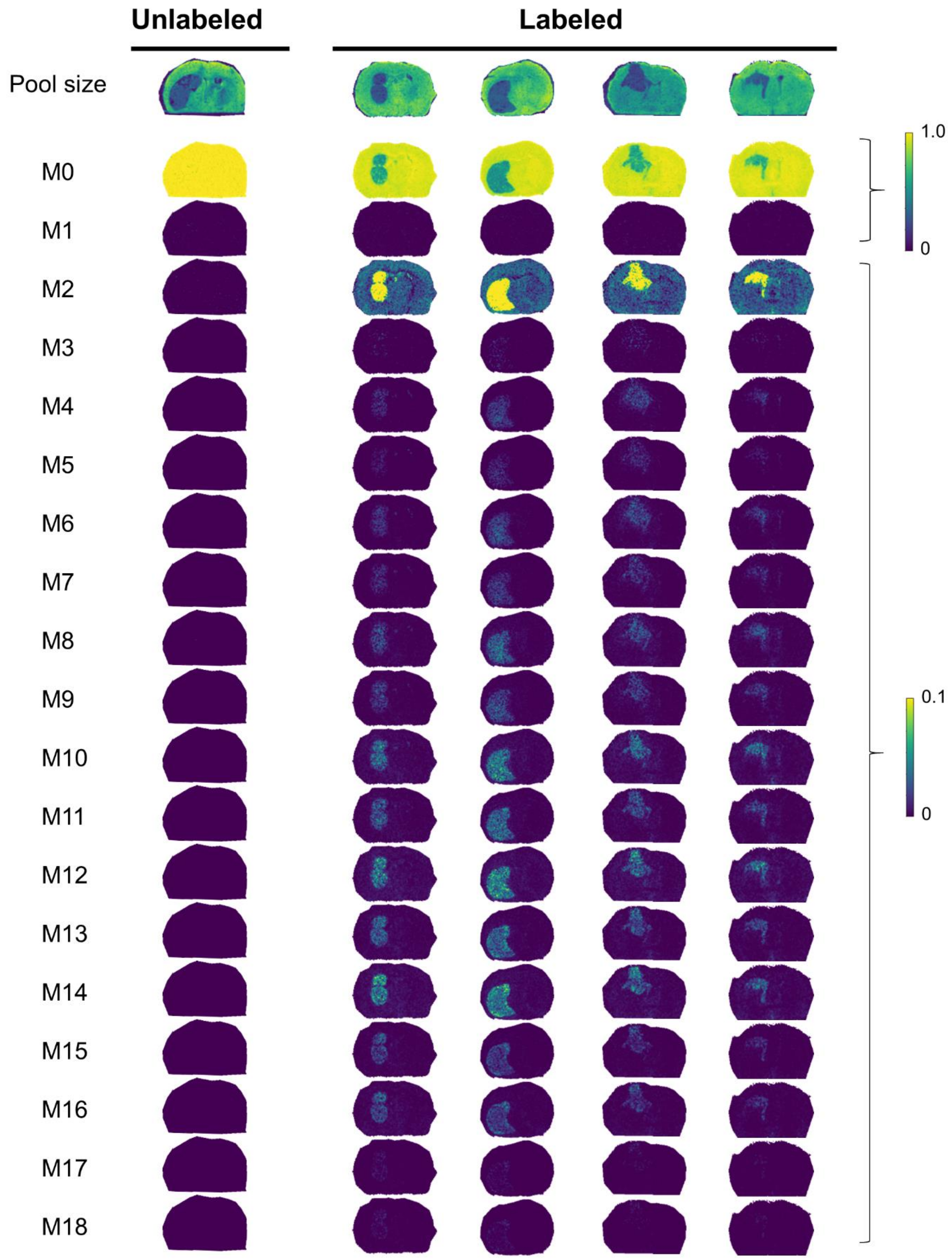

Supplementary Figure 15. Stearate isotopologue imaging with MALDI. Individual isotopologue images for palmitate in an unlabeled and four labeled tumor brains after correction for natural isotope abundance from MALDI data. Note the different intensity scale for M0 and M1 compared to the other isotopologues.

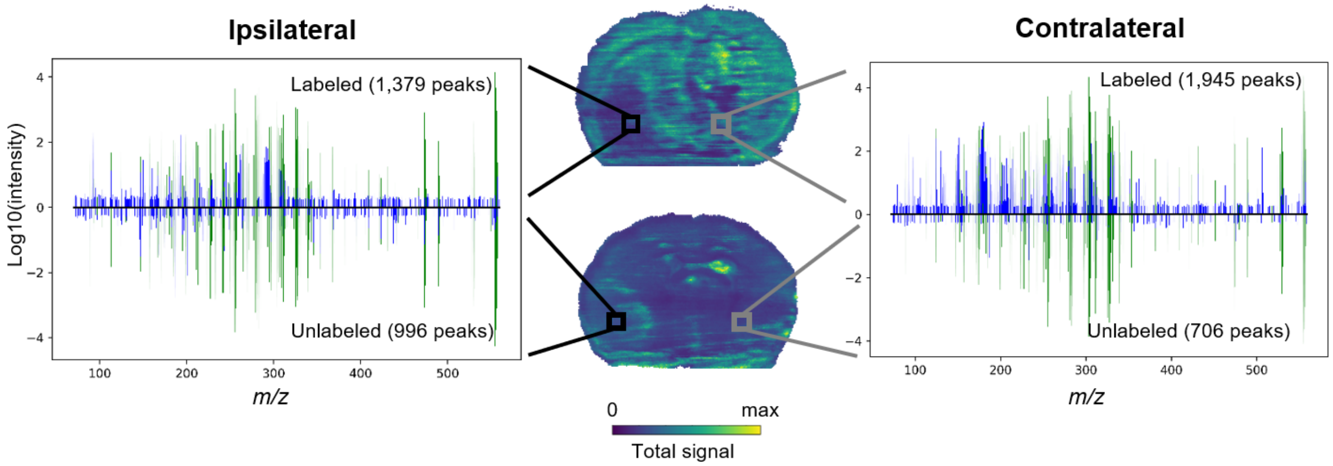

Supplementary Figure 16. Spectral comparison of unlabeled and labeled MSI data. Averaged mass spectra from two regions (ipsilateral and contralateral) of both a labeled (top) and unlabeled (bottom) brain analyzed by DESI MSI. Green peaks are shared between unlabeled and labeled spectra while blue peaks are unique. In both regions, introduction of isotopic label increases the complexity of the acquired spectra.

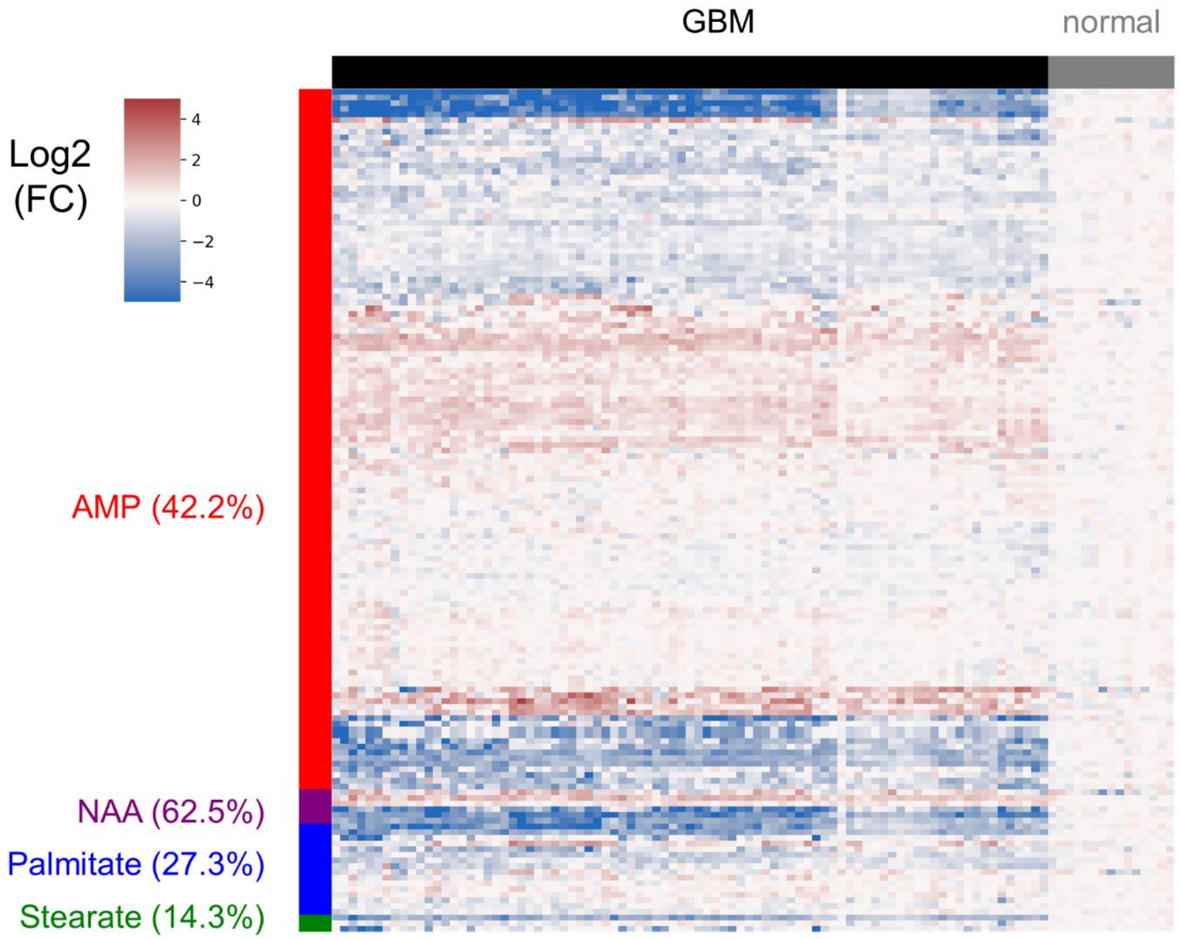

Supplementary Figure 17. Transcriptomics data from human samples. Many genes related to AMP, NAA, palmitate, and stearate are differentially expressed in brain samples from glioblastoma (GBM) patients versus normal brain samples. The percentage of genes with $p < 0.05$ (Wilcoxon rank-sum test, Bonferroni corrected) are shown in parentheses. Data are taken from Gene Expression Omnibus accession number GSE147352. NAA *N*-acetylaspartate, AMP adenosine monophosphate.

Supplementary Table 1. Metabolites detected and identified within the DESI MSI data. Metabolites were identified in the raw MSI data through natural abundance isotope pattern based matching in METASPACE. Compounds were manually curated and had a <10% false discovery rate.

Ion	Formula	Adduct	m/z	Name
C32H36N4O5-H-	C32H36N4O5	M-H	555.2612	Quinidine barbiturate
C33H36N2O6-H-	C33H36N2O6	M-H	555.2501	Dipiperamide
C14H28O2-H-	C14H28O2	M-H	227.2016	Myristate
C3H6O3-H-	C3H6O3	M-H	89.0243	Lactate
C17H34O2-H-	C17H34O2	M-H	269.2485	Nonyl octanoate
C15H30O2-H-	C15H30O2	M-H	241.2172	Dodecyl propionate
C19H22O3-H-	C19H22O3	M-H	297.1495	Gravelliferone
C21H26O3-H-	C21H26O3	M-H	325.1808	Isoacitretin
C16H32O2-H-	C16H32O2	M-H	255.2329	Palmitate
C10H14N5O7P-H-	C10H14N5O7P	M-H	346.0557	Adenosine monophosphate
C18H30O3S-H-	C18H30O3S	M-H	325.1842	Dodecylbenzenesulfonate
C17H28O3S-H-	C17H28O3S	M-H	311.1686	Undecylbenzenesulfonate
C20H38O7S-H-	C20H38O7S	M-H	421.2265	Methylacetophenone
C22H28O3-H-	C22H28O3	M-H	339.1965	Canrenone
C18H36O2-H-	C18H36O2	M-H	283.2642	Stearate
C20H26N2O2-H-	C20H26N2O2	M-H	325.1921	Ajmaline
C8H10O6-H-	C8H10O6	M-H	201.0404	Ethylaconitate
C27H46O4-H-	C27H46O4	M-H	433.3323	Dihydroxycoprostanate
C20H32O2-H-	C20H32O2	M-H	303.2329	Arachidonate
C6H9NO5-H-	C6H9NO5	M-H	174.0407	*N*-acetylaspartate

Supplementary Table 2. Metabolites detected and identified within the MALDI MSI data acquired with 12 matrix layers. Metabolites were identified in the raw MSI data through natural abundance isotope pattern based matching in METASPACE. Compounds were manually curated and had a <10% false discovery rate.

Ion	Formula	Adduct	m/z	Name
C10H14N5O7P-H-	C10H14N5O7P	M-H	346.0557	Adenosine monophosphate
C10H17N3O6S-H-	C10H17N3O6S	M-H	306.0765	Glutathione
C10H9N-H-	C10H9N	M-H	142.0661	6-Methylquinoline
C12H14N2-H-	C12H14N2	M-H	185.1083	Detomidine
C16H32O2-H-	C16H32O2	M-H	255.2329	Palmitate
C18H36O2-H-	C18H36O2	M-H	283.2642	Stearate
C20H32O2-H-	C20H32O2	M-H	303.2329	Arachidonate
C2H7NO3S-H-	C2H7NO3S	M-H	124.0073	Taurine
C4H12NO4P-H-	C4H12NO4P	M-H	168.0430	Phosphodimethylethanolamine
C4H6O5-H-	C4H6O5	M-H	133.0142	Malate
C4H7NO4-H-	C4H7NO4	M-H	132.0302	Aspartate
C5H10N2O3-H-	C5H10N2O3	M-H	145.0618	Glutamine
C5H9NO3-H-	C5H9NO3	M-H	130.0509	4-Hydroxyproline
C5H9NO4-H-	C5H9NO4	M-H	146.0458	Glutamate
C6H11O8P-H-	C6H11O8P	M-H	241.0118	Inositol cyclic phosphate
C7H12O3S-H-	C7H12O3S	M-H	175.0434	Methylthio 2-(propanoyloxy)propanoate

Supplementary Table 3. Metabolites detected and identified within the MALDI MSI data acquired with 14 matrix layers. Metabolites were identified in the raw MSI data through natural abundance isotope pattern based matching in METASPACE. Compounds were manually curated and had a <10% false discovery rate.

Ion	Formula	Adduct	m/z	Name
C10H17N3O6S-H-	C10H17N3O6S	M-H	306.0765	Glutathione
C10H9N-H-	C10H9N	M-H	142.0661	6-Methylquinoline
C2H7NO3S-H-	C2H7NO3S	M-H	124.0073	Taurine
C3H7O7P-H-	C3H7O7P	M-H	184.9856	Phosphoglycerate
C4H12NO4P-H-	C4H12NO4P	M-H	168.0430	Phosphodimethylethanolamine
C4H6O5-H-	C4H6O5	M-H	133.0142	Malate
C4H7NO4-H-	C4H7NO4	M-H	132.0302	Aspartate
C5H10N2O3-H-	C5H10N2O3	M-H	145.0618	Glutamine
C5H9NO3-H-	C5H9NO3	M-H	130.0509	Hydroxyproline
C5H9NO4-H-	C5H9NO4	M-H	146.0458	Glutamate
C6H11O8P-H-	C6H11O8P	M-H	241.0118	Inositol cyclic phosphate
C6H13O9P-H-	C6H13O9P	M-H	259.0224	Hexose phosphate
C6H9NO5-H-	C6H9NO5	M-H	174.0407	*N*-acetylaspartate
C7H12O3S-H-	C7H12O3S	M-H	175.0434	Methylthio 2-(propanoyloxy)propanoate
