# Supplementary material for: Using mass spectrometry imaging to map fluxes quantitatively in the tumor ecosystem
Source: Nat Commun. 2023 May 19;14:2876. doi: 10.1038/s41467-023-38403-x (PMC10199024; doi:10.1038/s41467-023-38403-x)
Supplement: Supplementary file 2 — Reporting Summary [file 41467_2023_38403_MOESM2_ESM.pdf]

nature portfolio

Corresponding author(s): Gary J. Patti

Last updated by author(s): Apr 7, 2023

Reporting Summary

Nature Portfolio wishes to improve the reproducibility of the work that we publish. This form provides structure for consistency and transparency in reporting. For further information on Nature Portfolio policies, see our Editorial Policies and the Editorial Policy Checklist.

Statistics

For all statistical analyses, confirm that the following items are present in the figure legend, table legend, main text, or Methods section.

n/a	Confirmed	
☐	☒	The exact sample size (*n*) for each experimental group/condition, given as a discrete number and unit of measurement
☐	☒	A statement on whether measurements were taken from distinct samples or whether the same sample was measured repeatedly
☐	☒	The statistical test(s) used AND whether they are one- or two-sided<br>*Only common tests should be described solely by name; describe more complex techniques in the Methods section.*
☒	☐	A description of all covariates tested
☐	☒	A description of any assumptions or corrections, such as tests of normality and adjustment for multiple comparisons
☐	☒	A full description of the statistical parameters including central tendency (e.g. means) or other basic estimates (e.g. regression coefficient) AND variation (e.g. standard deviation) or associated estimates of uncertainty (e.g. confidence intervals)
☐	☒	For null hypothesis testing, the test statistic (e.g. *F*, *t*, *r*) with confidence intervals, effect sizes, degrees of freedom and *P* value noted<br>*Give P values as exact values whenever suitable.*
☒	☐	For Bayesian analysis, information on the choice of priors and Markov chain Monte Carlo settings
☒	☐	For hierarchical and complex designs, identification of the appropriate level for tests and full reporting of outcomes
☒	☐	Estimates of effect sizes (e.g. Cohen's *d*, Pearson's *r*), indicating how they were calculated

Our web collection on statistics for biologists contains articles on many of the points above.

Software and code

Policy information about availability of computer code

Data collection	Data were acquired with mass spectrometry vendor software (Waters: HDI v1.5 and MassLynx V4.2, Bruker: Bruker Compass fleximaging 7.1 and timsControl 4.0.4, Thermo: Tune Application 3.3, Xcalibur 4.3).
Data analysis	Prism 9.3, HDI v1.5, Images were processed with the SIMSIToolBox software developed for this study (https://github.com/e-stan/imaging, v0.1.4) in Python 3.7.

For manuscripts utilizing custom algorithms or software that are central to the research but not yet described in published literature, software must be made available to editors and reviewers. We strongly encourage code deposition in a community repository (e.g. GitHub). See the Nature Portfolio guidelines for submitting code & software for further information.

Data

Policy information about availability of data

All manuscripts must include a data availability statement. This statement should provide the following information, where applicable:

- Accession codes, unique identifiers, or web links for publicly available datasets
- A description of any restrictions on data availability
- For clinical datasets or third party data, please ensure that the statement adheres to our policy

The MALDI and DESI MSI data were uploaded to METASPACE and are available at https://metaspace2020.eu/project/MSH_MSI_SISA_2023. LC/MS data were uploaded to the Metabolomics Workbench (project identifier: PR001630). The publicly available transcriptomics dataset is available at the Gene Expression Omnibus under accession number GSE147352. Source data for all figures are provided with this paper.

Human research participants

Policy information about studies involving human research participants and Sex and Gender in Research.

Reporting on sex and gender	N/A
Population characteristics	N/A
Recruitment	N/A
Ethics oversight	N/A

Note that full information on the approval of the study protocol must also be provided in the manuscript.

Field-specific reporting

Please select the one below that is the best fit for your research. If you are not sure, read the appropriate sections before making your selection.

[x] Life sciences   [ ] Behavioural & social sciences   [ ] Ecological, evolutionary & environmental sciences

For a reference copy of the document with all sections, see nature.com/documents/nr-reporting-summary-flat.pdf

Life sciences study design

All studies must disclose on these points even when the disclosure is negative.

Sample size	For isotopically labeled samples, n=4. For non-isotopically labeled samples, n=5. No statistical analysis was performed to determine these sample sizes. The sample sizes were selected based on suggestions from the reviewers and comparable studies of mouse brain metabolism.
Data exclusions	For aspartate and citrate, Grubbs' outlier test (α=0.05) identified one outlier sample. Thus, the plots in Figure 3c (aspartate) and Figure 7b (citrate) only show n=3 isotopically labeled samples. All other plots show data for n=4 isotopically labeled samples.
Replication	We verified the MSI results in four isotopically labeled mice. All attempts were successful.
Randomization	Samples were analyzed in a randomized order.
Blinding	We did not perform blinding as statistical comparisons (tumor/non-tumor) were only made within an animal. Tumor and non-tumor locations were confirmed with florescent microscopy.

Reporting for specific materials, systems and methods

We require information from authors about some types of materials, experimental systems and methods used in many studies. Here, indicate whether each material, system or method listed is relevant to your study. If you are not sure if a list item applies to your research, read the appropriate section before selecting a response.

Materials & experimental systems

n/a	Involved in the study
☒	☐ Antibodies
☐	☒ Eukaryotic cell lines
☒	☐ Palaeontology and archaeology
☐	☒ Animals and other organisms
☒	☐ Clinical data
☒	☐ Dual use research of concern

Methods

n/a	Involved in the study
☒	☐ ChIP-seq
☒	☐ Flow cytometry
☒	☐ MRI-based neuroimaging

Eukaryotic cell lines

Policy information about cell lines and Sex and Gender in Research

Cell line source(s)	GL261 murine glioma cells were a gift of the Milan G. Chheda laboratory and originally from the laboratory of Yancey Gillespie, University of Alabama at Birmingham, Birmingham, Alabama, USA
Authentication	All cells were verified by PCR amplification and Sanger Sequencing to confirm the IDH mutation at the genomic level.
Mycoplasma contamination	The cell line used was verified to be Mycoplasma negative.
Commonly misidentified lines (See ICLAC register)	None

Animals and other research organisms

Policy information about studies involving animals; ARRIVE guidelines recommended for reporting animal research, and Sex and Gender in Research

Laboratory animals	Female mice (C57BL/6J, eight weeks old) were obtained from Jackson Laboratory. Mice were housed with an ambient temperature of 22 degrees Celsius, a relative humidity of 55%, and a 12-h light/dark cycle.
Wild animals	No wild animals were used in this study.
Reporting on sex	Sex was not considered in study design.
Field-collected samples	No field collected samples were used in this study.
Ethics oversight	Animal experiments were approved by the Institutional Animal Care and Use Committee at Washington University (assurance number A338101, protocol 19-0930 and 22-0304) and were performed in accordance with the recommendations in the Guide for the Care and Use of Laboratory Animals of the NIH.

Note that full information on the approval of the study protocol must also be provided in the manuscript.
